# Methodological validation of Miro1 retention as a candidate Parkinson’s disease biomarker

**DOI:** 10.1038/s41531-025-01115-8

**Published:** 2025-09-15

**Authors:** Layla Drwesh, Giuseppe Arena, Daniel J. Merk, Daniele Ferrante, Vyron Gorgogietas, Thomas Gasser, Anne Grünewald, Patrick May, Kathrin Brockmann, Rejko Krüger, Richard Wüst, Christian Johannes Gloeckner, Julia C. Fitzgerald

**Affiliations:** 1 Department of Neurodegeneration, University Hospital Tübingen, Hertie Institute for Clinical Brain Research, Eberhard Karls University Tübingen, Tübingen, Germany; 2 Translational Neuroscience, Luxembourg Centre for Systems Biomedicine (LCSB), University of Luxembourg, Belvaux, Luxembourg; 3 Department of Neurology & Interdisciplinary Neuro-Oncology, University Hospital Tübingen, Hertie Institute for Clinical Brain Research, Eberhard Karls University Tübingen, Tübingen, Germany; 4 German Center for Neurodegenerative Diseases (DZNE), Tübingen, Germany; 5 Transversal Translational Medicine, Luxembourg Institute of Health (LIH), Strassen, Luxembourg; Parkinson Research Clinic, Centre Hospitalier de Luxembourg, Luxembourg, Luxembourg; 6 University Hospital Tübingen, Center for Mental Health, Eberhard Karls University Tübingen, Tübingen, Germany

**Keywords:** Neuroscience, Biomarkers

## Abstract

Mitochondrial markers help stratify Parkinson’s disease (PD) patients. We use high-throughput blotting to quantify Miro1, Mfn2, and VDAC levels in fibroblasts, blood cells, and iPSC-derived neurons. Miro1 is specifically retained in PD cells but degraded in healthy ones after mitochondrial depolarization. We correlate Miro1 retention scores with pathogenic mutations, genetic background, age, and clinical data. This scalable assay and quantifiable score for mitochondrial-PD support biomarker development and pharmacological screening.

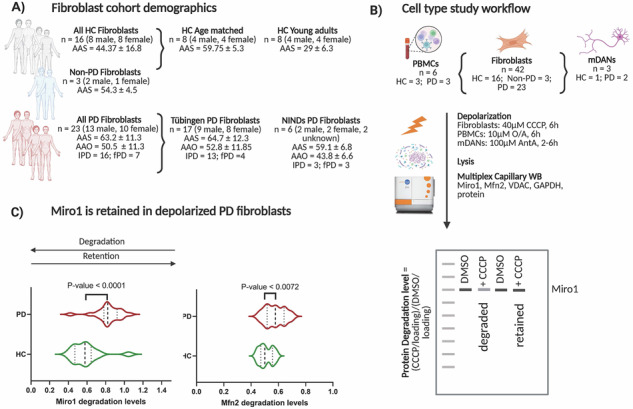

Early diagnosis and treatment of Parkinson’s disease (PD) remains challenging, underscoring the need for reliable diagnostic, prognostic and therapeutic biomarkers. Mitochondrial dysfunction is a key hallmark of PD, with genetic and environmental factors contributing to impaired mitochondrial quality control. Miro1, a mitochondrial outer membrane protein encoded by the *RHOT1* gene, plays a crucial role in mitochondrial transport along microtubules^1,2^, calcium homeostasis^3,4^, and mitophagy^5,6^. It interacts with several PD-associated proteins, including PINK1^7^ and LRRK2^8^. Upon mitochondrial damage, Miro1 was shown to be phosphorylated in the presence of PINK1^5^, which accumulates at the outer membrane, phosphorylating ubiquitin and the E3 ligase Parkin; leading to its activation and recruitment to the mitochondria. Parkin then ubiquitinates various outer membrane proteins, including Miro1^9,10^, marking them for degradation via the ubiquitin-proteasome system. This degradation is a crucial step in halting mitochondrial motility and initiating mitophagy, a selective process in which damaged mitochondria are engulfed and cleared by autophagosomes^11^. Recent genetic studies have linked *RHOT1* variants to PD, identifying rare heterozygous mutations that disrupt mitochondrial function, impair calcium homeostasis, and alter mitochondrial-ER contact sites^12,13^. However, genetic associations remain inconclusive, with some studies failing to establish a direct link^14–17^. Notably, large-scale rare variant burden testing ranked *RHOT1/*Miro1 among the top nominally significant genes in PD, suggesting a possible role in disease susceptibility^18^.

Given its involvement in PD-related pathways, Miro1 has emerged as a potential biomarker and target candidate for pharmacological intervention. A previous study demonstrated that the majority of PD fibroblasts (~94%) from both familial and idiopathic cases exhibited abnormal retention of Miro1 following mitochondrial depolarization^19,20^.

## Miro1 is specifically retained at depolarized mitochondria in PD fibroblasts

**Fig. 1:**
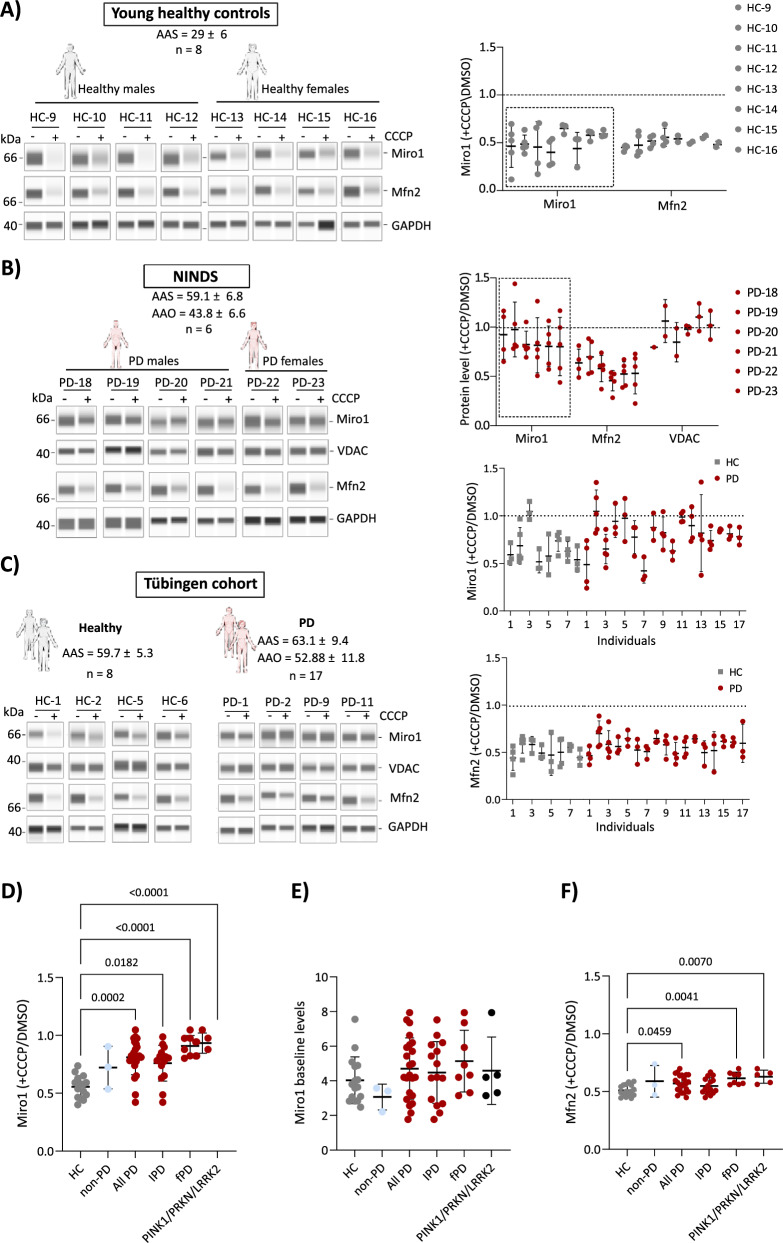
Significantly more retention of Miro1 in CCCP-treated fibroblasts from PD patients compared to healthy controls. **A–C** Whole-cell lysates from fibroblasts derived from (**A**) young healthy controls (HC), (**B**) Parkinson´s disease (PD) patients from the National Institute of Neurological Disorders and Stroke (NINDS) cohort and (**C**) an independent cohort of healthy individuals and PD patients from the Hertie Institute biobank (Tübingen cohort) were analyzed using simple western blotting (JESS, Bio-Techne). The mean age at sampling (AAS) and age at onset (AAO) ± standard deviation (SD) for each group are indicated. Fibroblasts from each cohort were seeded and treated simultaneously with either DMSO (vehicle control, -) or 40 µM CCCP for 6 h to induce mitochondrial depolarization. Miro1 protein levels were assessed under depolarizing conditions alongside control proteins, including mitochondrial markers (Mfn2 and VDAC) and the cytosolic housekeeping protein GAPDH (loading control). Protein intensities were quantified using CompassForSW software and normalized to GAPDH within the same lane. The response to depolarization was calculated as the ratio of protein levels in CCCP-treated samples relative to vehicle controls (CCCP/DMSO). The average CCCP-response value for each protein was determined from at least three biological replicates. Scatter-plot graphs display the average CCCP-response value for each protein, based on at least three biological replicates. Error bars represent the standard deviation (SD) for each individual. **D–F** Protein average levels are calculated per each individual and represented by scatter plot in each group of the following: Healthy controls (HC) *n* = 16, non-PD (Ataxia, FTD, and ALS) *n* = 3, all Parkinson´s disease patients (PD) *n* = 23, idiopathic PD (iPD) *n* = 16, familial PD (FPD) *n* = 8, non-pathogenic variants (NPV) *n* = 11 and pathogenic variants *n* = 5. **D** Miro1 depolarization-response values. **E** Miro1 baseline levels **F** Mfn2 depolarization-response values. The average values are compared between different groups. Error bars indicate the standard deviation between different individuals within each group. Statistical significance of the CCCP-response values between different groups was assessed using one-way ANOVA test, with *P* values <0.05, stated numerically on the graph.

Similar to the previous publication^19^, we employed a mitochondrial depolarization assay to assess Miro1 response in fibroblasts. In fibroblasts from healthy individuals (age at sampling (AAS)_avg_ = 29 ± 6.1 y), we observed clear and consistent degradation of both Miro1 and Mitofusin2 (Mfn2) following 6 h of 40 µM CCCP treatment (Fig. 1A). Inhibition of Miro1 and Mfn2 degradation upon MG132 treatment under depolarization conditions confirms the proteasome-specific degradation (Fig. S1A). We report independent validation of an overall Miro1 retention phenotype in the same PD patient group used by Hsieh and colleagues^19^ (Fig. S1B). We analyzed fibroblasts from an independent Tübingen cohort containing healthy age- and sex- matched controls (HC) and PD patients, alongside three non-PD controls (diagnosed with Ataxia, FTD-Frontotemporal Dementia and ALS-Amyotrophic Lateral Sclerosis). Each experiment was replicated independently (the full dataset is shown in Figs. S2 and S3 and collection of a representative four healthy controls and four PD patients with the overall Miro1 and Mfn2 quantification data is shown in Fig. 1C. Overall, healthy fibroblasts degraded Miro1 and Mfn2 in response to CCCP, whereas PD fibroblasts specifically retained Miro1 (Fig. 1C).

VDAC levels were unchanged (Fig. S1C, D) which is in line with previous studies suggesting that degradation of VDAC occurs at later stages after CCCP-induced depolarization^21^. The coefficient of variance (CoV%) analysis of Miro1, Mfn2 and VDAC degradation levels revealed similar variability between HC and PD groups (Fig. S1G).

Non-PD controls exhibited Miro1 degradation (FTD and ALS) and mild Miro1 retention (Ataxia) but more numbers are needed (see Fig. 1D and Figs. S2A and S3A). Taken together, all PD groups have significantly more Miro1 retention compared to healthy controls (Fig. 1D), with the majority of PD patients and IPD patients exhibiting similar Miro1 retention (Ratio 0.8 ± SD 0.15) *P* < 0.005. Fibroblasts from familial PD patients (Ratio 0.89 ± SD 0.1) *P* < 0.0001, including those with PINK1/PRKN/LRRK2 mutations (Ratio 0.93 ± SD 0.1) *P* < 0.0001 exhibited the strongest Miro1 retention ratio in contrast to healthy controls (Ratio 0.5 ± SD 0.1) (Fig. 1D). Miro1 steady state levels at baseline were not significantly different to the control group (Fig. 1E) nor was the Mfn2 and the VDAC ( + CCCP/DMSO) ratio (Fig. 1F, Fig. S1C).

To overcome issues of sensitivity and specificity in the assay, we optimized for a fully automated JESS Simple Western™ system (Bio-Techne)^22^. Miro1 antibodies were tested (Fig. S4A) and subjected to pre-clearing assays (Fig. S4B) and Miro1 knockdown in fibroblasts (Fig. S4E, F).

## PINK1/PRKN PD fibroblasts retain Miro1 even after long-term mitochondrial depolarization

**Fig. 2:**
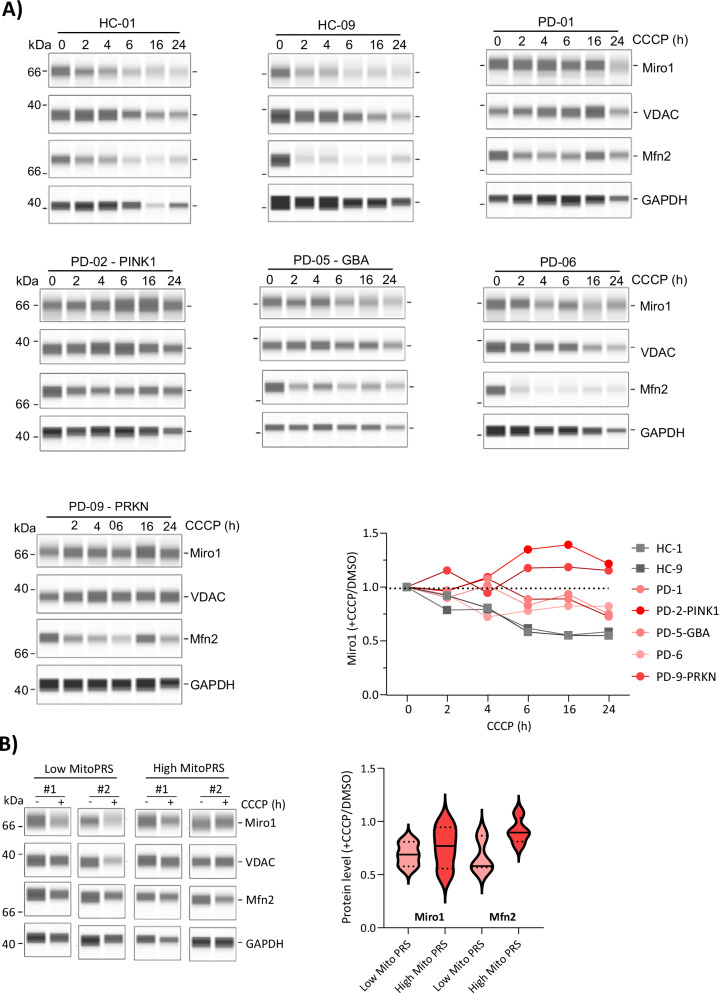
Miro1 degradation is retarded in IPD but inhibited in PINK1/PRKN PD. **A** Fibroblast cell lines from two healthy controls and five PD patients were treated with 40 µM CCCP for the indicated time points to assess Miro1 degradation over time. Miro1 protein levels were quantified using CompassForSW software, and its response to depolarization was expressed as the ratio of treated to vehicle control (time point 0). **B** Fibroblast cell lines from two PD patients with low mitochondria-specific polygenic risk score (mitoPRS) and two PD patients with high mitoPRS were treated with 40 µM CCCP for 6 h. Miro1 depolarization response was measured using CompassForSW software and compared to Mfn2 depolarization response as show in the graph. Statistical significance of the CCCP-response values between low Mito-PRS and high Mito-PRS was assessed using one-way ANOVA test, with *P* values stated numerically on the graph.

We measured the steady state level kinetics for Miro1, Mfn2, VDAC and GAPDH over a time course of CCCP treatment up to 24 h. In healthy cells, we observed degradation of Miro1 at 2 h proceeding towards ~0.5 Miro1 ( + CCCP/DMSO) by 6 h (Fig. 2A). Miro1 levels in IPD cells remained near baseline until approximately 6 hours post-treatment, after which a moderate decrease to ~0.75 ( + CCCP/DMSO) was observed (Fig. 2A). PD patients carrying PINK1 and PRKN mutations (see Table S1, Figs. S2 and S3) had unchanged Miro1 levels from baseline for the first 4 hours of CCCP treatment and then an increase to ~1.2 ( + CCCP/DMSO) (Fig. 2A). We analyzed four IPD fibroblast lines that had previously been genotyped and assigned a mitochondrial polygenic risk score (MitoPRS) by Arena and colleagues^23^. Those IPD fibroblasts with low MitoPRSs had a combined average 0.69 Miro1 retention ( + CCCP/DMSO) and those with high MitoPRSs had a combined average of 0.75 Miro1 retention (Fig. 2B).

**Fig. 3:**
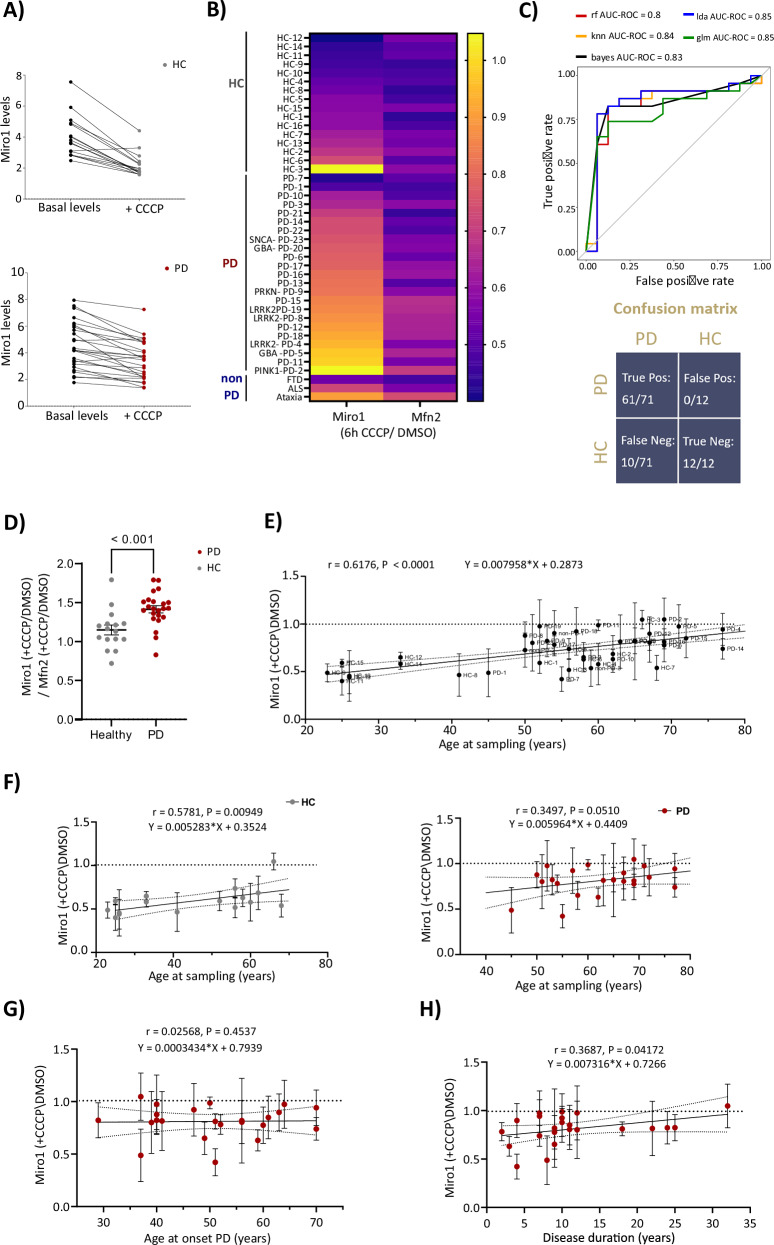
Miro1 degradation is linked to aging and disease duration. **A** Flux graph illustrating Miro1 levels in fibroblasts from healthy controls (HC-upper graph) and PD patients (lower graph) under basal (DMSO) and CCCP-treated conditions. Each line represents an individual, with the slope indicating the rate of Miro1 degradation over 6 h of CCCP treatment. Miro1 band intensities were normalized to GAPDH levels from the same lane. Data points represent mean values from at least three independent experiments (*n* ≥ 3). **B** Heatmap displaying the degradation levels for Miro1 and Mfn2 ( + CCCP/DMSO), in each individual across HC, PD and non-PD cohorts. **CUpper graph-** Receiver Operating Characteristic (ROC) curves based on our study dataset, where machine learning models were trained using our study´s Miro1 degradation values in the HC and PD groups. The trained models were then used to predict Healthy Controls (HC) and Parkinson’s disease (PD) individuals from Hsieh et al. 2019 study. Area Under the Curve (AUC-ROC) values indicate the discriminative ability of each model. Models compared: random forest (rf), K-nearest neighbors (knn), naïve Bayes (bayes), linear discriminant analysis (lda), and generalized linear model (glm). **Lower graph** Confusion matrix summarizing classification performance, showing the number of correctly classified cases (true positives and true negatives) and misclassified cases (false positives and false negatives). **D** Comparison of Miro1 degradation relative to Mfn2 degradation in response to CCCP treatment across individuals in HC and PD groups. Data are presented as scatter-plot with mean ± SEM. Statistical significance was assessed using Mann-Whitney test, with P-values stated numerically on the graph. **E** Correlation between Miro1 average retention levels ± SD, and age at sampling (AAS) across all individuals and cohorts. **F** Correlation between Miro1 retention levels ± SD and age at sampling, analyzed separately for HC (upper graph) and PD (lower graph) groups. **G** Correlation between Miro1 retention levels ± SD and age at onset (AAO) is represented across individuals in PD group. **H** Correlation between Miro1 retention levels ± SD and disease duration (years since PD diagnosis) across individuals in PD group. **F–H** Pearson correlation (r) and Linear regression analysis were performed.

There are no differences in baseline levels of Miro1 in PD patients (Fig. 1E). We plotted Miro1 levels at baseline and then at the 6 h + CCCP endpoint (Fig. 3A). With one exception (HC-3), all HC fibroblasts in this study showed normal Miro1 degradation. The healthy donor HC-3 was not followed as part of a longitudinal study. In the PD group, steady state levels of Miro1 are overall reduced after 6 h of CCCP treatment (Fig. 3A). The mean depolarization response of Miro1 and Mfn2 for each individual across all fibroblast cohorts is visualized by a heatmap (Fig. 3B). We applied several machine learning algorithms trained on the Tübingen dataset, where we converted the Miro1 +CCCP/DMSO ratios to % inhibition of Miro1 degradation. We asked the machine to predict whether the % inhibition scores from the Hsieh et al. 2019 dataset were from a healthy person or a PD patient. Each machine learning model was assessed using 10-fold cross-validation, with performance metrics (Fig. S1H), and Receiver Operating Characteristic – Area under the curve (ROC-AUC) (Fig. 3C**upper graph**). The accuracy and Kappa statistics (Fig. S1H) further affirmed the reliability of these models in making predictions. Disease prediction outcome is shown as a confusion matrix (Fig. 3C**lower graph**). This indicates high sensitivity (61/71 true positives) and specificity (0 false positives) to differentiate PD from HC.

## Miro1 retention increases with age in healthy people but not PD patients

Similar to Miro1, Mfn2 is a substrate of the PINK1/PRKN pathway; however, it is not known to be a substrate of LRRK2 as has been suggested for Miro1^24^. Fig. 3D compares Mfn2 and Miro1 degradation levels in PD and HC groups. The age of donors at skin biopsy (AAS) was also considered, given that autophagy is known to decline with age^25^. Across both PD (AAS_avg_ = 63.2 ± 11.3 y, 45–77 y) and HC (AAS_avg_ = 44.3 ± 16 y, 25–68 y) groups, AAS showed a significant positive correlation with Miro1 retention (*r* = 0.6176, *P* < 0.0001), with an estimated yearly increase of 0.007958 (Fig. 3E). When analyzed separately, the HC group demonstrated a moderate significant correlation (*r* = 0.5781, *P* = 0.0095), whereas the PD group showed a weaker, non-significant correlation (*r* = 0.3497, *P* = 0.0519) (Fig. 3F), indicating that the increased Miro1 retention observed in PD is not an AAS effect. There was no significant association between age at disease onset (AAO) and Miro1 retention (*r* = 0.02568, *P* = 0.04537) (Fig. 3G). In contrast, disease duration showed a moderate statistically significant positive correlation with Miro1 retention (*r* = 0.3687, *P* = 0.0417) (Fig. 3H). To address differences in age distribution between HC and PD groups, we reanalyzed a subset with a matched AAS range (Fig. S1I). Additionally, no significant effect of sex on Miro1 retention was observed in either the HC or PD group (Fig. S1E). Of those PD patients in this study where cerebral spinal fluid (CSF) alpha synuclein seeding data was available (PMID: 34717775, PMID: 38242875), the average Miro1 ( + CCCP/DMSO) retention was higher in SAA negative than SAA positive seeders but was not statistically significant (Figure S1F).

## Miro1 retention can be observed in blood cells and human dopaminergic neurons

**Fig. 4:**
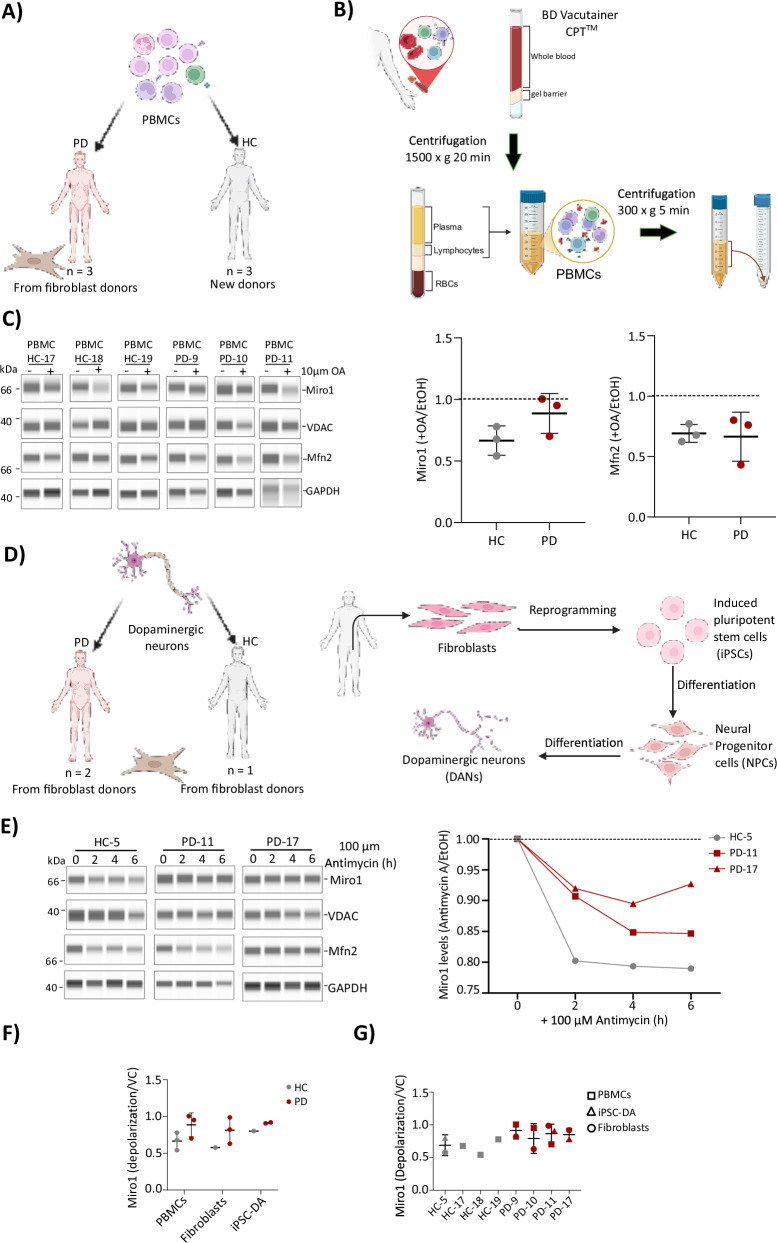
Miro1 is retained following depolarization of mitochondria in peripheral blood cells and dopaminergic neurons from PD patients that had previously donated a skin biopsy. **A** Peripheral blood mononuclear cell lines (PBMCs) from a total of six donors, including three healthy controls (HC) and three Parkinson’s disease (PD) patients were included in our study. The PBMCs PD donors were the same individuals in the fibroblast cohort (PD-9, PD-10, PD-11). **B** PBMCs were isolated from blood samples collected using BD Vacutainer® CPT™ tubes. Whole blood was centrifuged to separate plasma from the PBMC layer, which was then carefully transferred, washed, and cryopreserved for downstream analyses. **C** Whole-cell lysates of PBMCs were analyzed using simple western blotting (JESS, Bio-Techne) to assess Miro1 response following mitochondrial depolarization with 10 µM oligomycin and 10 µM antimycin (OA) for 6 h. Protein intensities were quantified using CompassForSW software and normalized to GAPDH within the same lane. The response to depolarization was calculated as the ratio of protein levels in oligomycin and antimycin- treated samples to those in the vehicle control - ethanol treated samples ( + OA/EtOH). Distribution of Miro1 (left graph) and Mfn2 (right graph) depolarization responses across individuals in the HC group (gray, *n* = 3) and PD group (red, *n* = 3). **D** Dopaminergic neuron (DAN) cell lines were generated from fibroblasts of three donors, including one healthy control and two sporadic Parkinson’s disease (PD) patients. The fibroblasts were reprogrammed into induced pluripotent stem cells (iPSCs), which were subsequently differentiated into neural progenitor cells (NPCs) and further matured into dopaminergic neurons. The three DANs lines were generated from individuals in our fibroblast cohort (HC-5, PD-11, PD-17). **E** WCL of DANs were analyzed using simple western blotting (JESS, Bio-Techne) to assess Miro1 response following mitochondrial depolarization with 100 µM antimycin for different time points (2, 4 and 6 h). Protein intensities were quantified using CompassForSW software and normalized to GAPDH within the same lane. (**F-G**) Distribution of Miro1 depolarization-response in HC individuals and PD patients across different cell types.

We next retrieved peripheral blood mononuclear cells (PBMCs) and induced pluripotent stem cells (iPSCs) from donors. For PBMCs, additional HC donors were sex matched to PBMCs collected from PD-9, PD-10 and PD-11 (Fig. 4A). A schematic depiction is shown in Fig. 4B. We treated PBMCs with or without 10 µM oligomycin and antimycin A for 6 h (Fig. 4C). There was a Miro1 ( + OA/EtOH vehicle) score of 0.66 for HC (higher than fibroblast HC average of 0.5) and 0.88 (similar to IPD fibroblasts) for the PD donors (Fig. 4C). There was no difference between HC and PD groups for the Mfn2 control (Fig. 4C), maintaining the Miro1 specific phenotype in blood as well as fibroblasts. In parallel we differentiated dopaminergic neurons from one HC and two PD patients (Fig. 4D). In healthy dopaminergic neurons (HC-5), we observed degradation of Miro1. A drop from 1.0 to ~0.8 Miro1 ( + AntA/EtoH vehicle) by 2 h which remains at a steady state throughout the 6 h time course. Mfn2 levels in HC followed a similar pattern to Miro1 (Fig. 4E). Miro levels in PD-11 and PD-17 (IPD) on average show less degradation than the control at ~0.9 at 2 h and then remain until 6 h (Fig. 4E). Miro1 is retained following mitochondrial depolarization in PD patient fibroblasts, PBMCs and dopaminergic neurons (Fig. 4F, G).

## Chemical induction of Miro1 degradation in fibroblasts

**Fig. 5:**
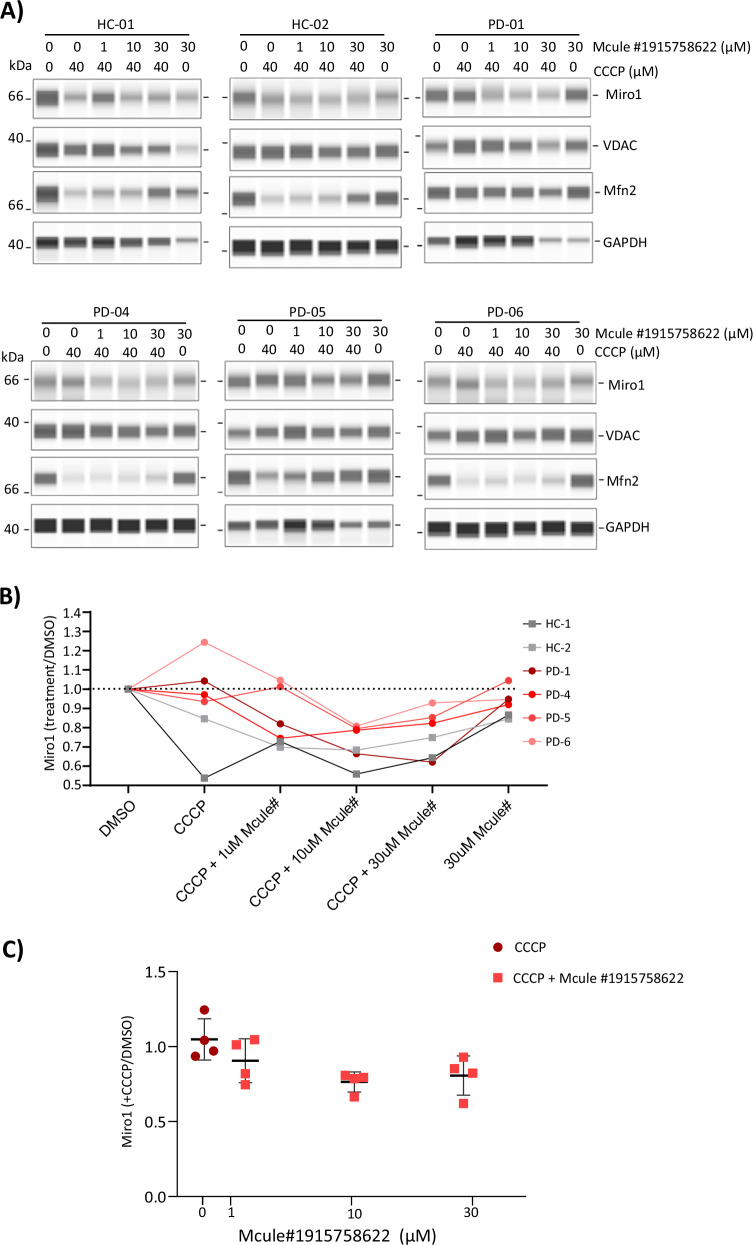
Compound #1915758622 only minimally improved the Miro1 phenotype observed in PD fibroblasts. **A** Fibroblasts from two healthy controls and four PD patients were used to assess the effect of compound 3 (Mcule #1915758622) on Miro1 retention upon mitochondrial depolarization. Cells were pretreated with varying concentrations of the compound (1, 10, and 30 µM) for 24 h, followed by 6 h treatment with 40 µM CCCP. A compound-only control (30 µM, no CCCP) was included for each cell line. **B** Graph showing Miro1 response to depolarization upon compound 3 pretreatment across different cell lines. Miro1 degradation levels were quantified using CompassForSW software and expressed as the ratio of treated to vehicle control (treatment/DMSO). **C** Distribution of Miro1 degradation levels in PD cell lines under different compound pretreatment concentrations. Statistical significance of the compound´s reducing potential of Miro1 retention at different concentrations was assessed using Friedman test (not significant, *P* > 0.05).

Hsieh et al. found 3-(3-fluorophenyl)-1-(4-{[6-(1 H-imidazol-1-yl)pyrimidin-4-yl]amino}phenyl)urea, a complex aminopyrimidine compound (referred to as Compound 3 or C3)^19^ to promote Miro1 degradation. Referred in this study as Mcule #1915758622), fibroblasts were pretreated with varying concentrations of the compound for 24 hours, followed by 6-hour treatment with CCCP (Fig. 5C). The effect of Mcule #1915758622 on Miro1 degradation following mitochondria depolarization (CCCP treatment) is illustrated in (Fig. 5B). 1 µM of the compound was sufficient to initiate Miro1 degradation in most of the PD fibroblast lines. At 10 µM, Miro1 was reduced to a degree comparable to those observed in healthy control lines. Reduced Miro1 was seen at 30 µM (Fig. 5A) but the differences compared to CCCP-only treatment were not significant (*P* > 0.05).

## Future perspectives and link to mitochondrial polygenic risk scores

In our analysis, we utilized the mean average of Miro1 signal at baseline and following CCCP induction to calculate a ratio reflecting Miro1 degradation under depolarizing conditions. While assay and technical variability were relatively low, and cell lines generally performed consistently across experiments, we observed significant intra-individual differences in the Miro1 retention ratio. We propose that Miro1 retention values (+depolarizing agent/vehicle control) could be assigned as an indicator of Miro1-mediated mitochondrial quality control with 0.55 being the mean average healthy score, ~0.8 average Miro1 score for IPD and >1.0 as Miro1-mediated mitophagy inhibition and in the range of familial PD and *PINK1/PRKN/LRRK2* PD.

The invasive nature of skin biopsies poses another limitation, underscoring the need for method translation to blood cells to facilitate the inclusion of much larger cohorts, inclusion of more non-PD controls and individuals at risk to develop PD is needed.

Although the method can be easily implemented in other laboratories using fibroblasts or other cell types, capillary Western blotting is not a standard technique. For high-throughput cohort studies, capillary Western blotting is advantageous because of direct and accurate quantification, low volumes, multiplexing, and costs are relative to traditional Western blot due to the low volumes of antibody used. We therefore shared detailed open access protocols for Capillary and traditional Western blot analysis on protocol.io (10.17504/protocols.io.e6nvwe5z9vmk/v1).

In this study, we include all known pathogenic mutations or known mutations and variants (non-pathogenic or of unknown significance) in PD-associated genes in Table S1. We provide the available polygenic risk scores (PRS) for the entire genome, mitochondrial (MitoPRS) and Lysosomal (LysoPRS) in Table S1 with annotation. Interestingly, HC-2 has an above average Miro1 retention score (0.68) for a control individual. The polygenic risk scores (PRS) suggest a high risk (top 20 percentile) in general MitoPRS, Mitophagy MitoPRS and Lysosomal protein catabolic process score. A familial PD patient with a GBA exon 10 duplication has a high Miro1 retention score (0.974), a high (top 20 percentile) whole genome PRS and MitoPRS and has a very high (top 10 percentile) MitoPRS for the Mitochondrial protein import. PD-7 (IPD) is a patient with a very low Miro1 retention score (0.421) below the mean average control score (0.55). Whole genome PRS and MitoPRS are in the lowest percentiles. Instead, the LysoPRSs are relatively high (top percentiles) for this individual. Therefore, future studies integrating genetic data in a larger dataset would be valuable for exploring the contribution of such variants to the observed phenotypes and for better stratifying patients.

## Methods

### Informed consent

Research involving human research participants, material, or data has been performed in accordance with the Declaration of Helsinki. Fibroblasts and PBMCs were donated to the Neuro-Biobank of the Hertie Institute for Clinical Brain Research, University of Tuebingen, Germany (https://www.hih-tuebingen.de/en/about-us/core-facilities/biobank/) following a skin biopsy that was agreed following informed consent. This biobank is supported by the University of Tübingen, the Hertie Institute, and the DZNE. Informed consent for fibroblast donation is approved by the ethics commission under standard consent forms archived at The Neuro-Biobank, Tübingen. All bio-samples are anonymized by an HIH ID number and basic data about sex, age at sampling is available via the internal RedCap system. Generation of iPSCs and subsequent differentiation has been approved by the ethics commission of The Medical Faculty of The University of Tübingen. Given the unique and limited nature of the materials, only a specified number of samples were provided. All fibroblast cell lines in this study are summarized in **Supplementary data** Table 1, which also contains the six fibroblast cell lines that were purchased from the NINDs human cell and data repository (https://nindsgenetics.org/). NINDS data is also anonymized by a NINDS ID number. Fibroblasts donated by young, healthy individuals used in this study also gave informed consent and were collected by Richard Wüst MD (Department of Psychiatry, Tübingen Center for Mental Health (TüCMH)) and were bio-banked according to the standard consent and procedures of the Neuro-Biobank of the Hertie Institute for Clinical Brain Research. Reasonable requests for further information and/or samples can be made to The Neuro-Biobank Tübingen or corresponding author.

### Culture of human fibroblasts

Fibroblasts were seeded from cryovials and maintained in a 37 °C, 5% CO2 incubator with humidified atmosphere and cultured in high glucose (4.5 g/L) DMEM (Sigma #D6429) containing 10% fetal bovine serum, supplemented with 1% non-essential amino acids (Thermofisher #11140035) and 1% penicillin-Streptomycin (Sigma-Aldrich #P0781). The plates were regularly examined, and the medium was replaced every 3–4 days until fibroblasts reached confluency. Confluent fibroblasts were trypsinized and transferred to a flask for further growth, with subsequent expansion into multiple dishes or flasks at a 1:4 split ratio. For storage, batches of ~2.5 × 105 cells per 0.5 ml of growth medium with 10% DMSO were frozen in cryo-vials. The temperature was gradually decreased before storage in liquid nitrogen by placing the vials in a freeing box containing isopropanol in a −80 °C freezer overnight up to 1 week before placing in liquid nitrogen container for long term storage.

### PBMC collection and storage

Immediately following blood draw and collection in three BD Vacutainer CPT^TM^ vials, the vials containing around 8–10 mL of blood were processed in the laboratory within 2 h of the blood draw. The vials are inverted gently 8–10 times and centrifuged at 1700–1500 × g for 20 min at room temperature in a swinging-bucket rotor. After centrifugation, the vials are inverted again 8–10 times. The plasma supernatant containing PBMCs was transferred into new 50 mL tube, washed once with PBS, and centrifuged at 300 × g for 5 min. The supernatant was carefully aspirated and the pellet containing PBMCs and remaining red blood cells (RBCs) was resuspended with 5–7 mL Erylyse buffer (0.1 mM EDTA, 155 mM NH_4_Cl, 10 mM KHCO_3_, pH 7.4) and incubated for 5 min to lyse RBCs. Cells were then washed with PBS and centrifuged at 300 × g for 5 min. PBMCs pellet was resuspended in 90% fetal bovine serum (FBS) + 10% DMSO and stored at −80 °C, followed by liquid nitrogen storage 24 h later. Cell viability and concentration were assessed by Trypan Blue using TC10 automated cell counter (Biorad). Where possible, 4–5 × 10^6^ cells were stored per cryovial.

### Culture of neural precursor cells (NPCs) and differentiation to dopaminergic neurons (hDaNs)

NPCs were derived from iPSCs as previously described^26^. NPCs were cultures in NPC maintenance medium, containing; base medium; (50% DMEM/F12 (Thermo Fisher Scientific (Waltham, MA, USA), #11-330-057), 50% neurobasal (Thermo Fisher Scientific (Waltham, MA, USA), 21103-049), 1% penicillin/streptomycin (Merck (Darmstadt, Germany), #A2213), 1% GlutaMax (Thermo Fisher Scientific (Waltham, MA, USA), #35050-038), 1% B27 supplement (without vitamin A; Thermo Fisher Scientific (Waltham, MA, USA), #12587-010), and 0.5% N2 supplement (Thermo Fisher Scientific (Waltham, MA, USA), #17502-048)) supplemented with 3 µM CHIR 99021, 200 µM ascorbic acid (Sigma-Aldrich (St. Louis, MO, USA), #A4544-25G), and 0.5 µM PMA) and plated on Matrigel (Corning (Corning, NY, USA), #354230). After several passages, NPCs were differentiated into dopaminergic neurons as previously described^27^, with the alteration that NPCs were not primed by removing the PMA from the NPC media prior to differentiation.

### Mitochondrial depolarization

#### Fibroblasts

Cells were divided into batches of 3–4 age- and sex-matched lines, each batch containing one healthy control and 2–3 PD or non-PD lines. Each cell line within a batch underwent identical handling, from cell seeding and treatment to cell lysing and subsequent analysis by Western blotting. Each cell line within the same batch was split to three wells in either 6 or 12 well-plate at a similar density prior to treatment. CCCP (C2759, Sigma-Aldrich) was prepared at 40 mM in DMSO and small volume aliquots were stored in −80 °C. 40 µM final CCCP concentration was applied to cell lines in fresh culture medium (high glucose DMEM, 10% fetal bovine serum, 1% non-essential amino acids and 1% penicillin-Streptomycin) at 1:1,000 dilution. The wells were treated as follows: first well: treated with DMSO (vehicle control), second well: treated with 40 µM of CCCP for 6 h to induce mitophagy by depolarization and third well: treated with 10 µM of the proteasome inhibitor MG132 in addition to 40 µM CCCP.

#### PBMCs

Human PBMCs were thawed in a 37 °C water bath, transferred to 15 mL of RPMI-1640, and centrifuged at 300 × g. The cell pellet was resuspended in RPMI-1640 with 10% FBS, 1% penicillin-streptomycin, and 1:1000 Apoi, then seeded in a non-coated Petri dish and incubated overnight. The next day, cells were collected, centrifuged, and seeded in 6-well plates (3–4 million cells per well). After 2 h, cells were treated with 10 µM Oligomycin and 10 µM Antimycin in one well, and EtOH vehicle control in the other, for 6 h. After treatment, cells were collected, washed with PBS, centrifuged, and either stored at −20 °C or immediately lysed in 20 µL lysis buffer (100 mM Tris, 150 mM NaCl, 1 mM EGTA, 1% Triton X-100, 0.5% sodium deoxycholate, 1 mM PMSF, 1:10 protease inhibitor cocktail).

#### hDaNs

Dopaminergic neurons at day 16–19 were treated for 0, 2, 4, 6, or 24 h with a final concentration of 100 µM Antimycin A in the neuronal maturation medium. Untreated hDANs received fresh neuronal maturation medium with EtOH (vehicle control) for 24 h.

### Preparation of cell lysates

Following treatment, cells were collected from the well by; trypsinization (fibroblasts), collection (PBMCs) and Accutase (hDaNs), and washed once with PBS, followed by centrifugation for 5 min at 300 x g. The cell pellet was then resuspended with 20–50 µl lysis buffer ((100 mM Tris, 150 mM NaCl, 1 mM EGTA, 1 mM EDTA, 1% Triton X-100, 0.5% sodium deoxycholate, 1 mM PMSF, 1:10 protease inhibitor cocktail (539134, Calbiochem, San Diego, United States of America))), and was either stored in −80 °C until further treatment or proceeded with 15–30 min incubation on ice to enable cell lysing. Supernatant of Whole cell lysates (WCL) were then collected after centrifugation for 15–30 min at 18,000 x g 4 °C. Protein estimation was performed using Pierce BCA Protein Assay Kit (Thermofisher, #23225). All lysates in the same batch were diluted using lysis buffer to have the same final concentration (typically between 1–2 mg/mL). WCL of cell lines from the same batch were then analyzed side by side using JESS Simple Western™ (Bio-Techne).

## Automated western blotting

WCL samples were analyzed on the JESS Simple Western^TM^ instrument (ProteinSimple®, Bio-Techne) using the 12–230 kDa separation module, 8 × 25 capillary cartridge (#SM-FL004 ProteinSimple®, Bio-Techne). DTT, biotinylated ladder and the fluorescent standard powder stocks were provided with the separation module (EZ standard pack I #PS-ST01EZ-8) and were dissolved in ddH_2_O according to the manufacturer’s protocol. Lysate samples were mixed with 1x fluorescent master mix, and diluted to final concentration of 0.4–0.8 mg/mL using 0.1x sample buffer (from 10x stock solution #042-195 ProteinSimple®). Prior to loading, diluted samples were heated for 5 min at 95 °C to denature proteins, and 4 µL (1.6–3.2 µg) was added per well. The following antibodies with the indicated dilutions were used for analyzing lysates after the depolarization assay: Miro1 (#HPA01687, Merck) at 1:10, Mfn2 (#H00009927, Abnova) at 1:50, GAPDH (#CB1001, Merck) at 1:1,000, and VDAC (#AB10527, Millipore) at 1:1,000. Each sample/capillary was decorated with all four antibodies using multiplexing and replexing modes, to this end, two probes were used with RePlex^TM^ reagent kit (#RP-001, ProteinSimple®) to allow sequential detection: in probe 1, Miro1 and VDAC primary antibodies were multiplexed and both were decorated with ready to use goat anti-rabbit secondary HRP-conjugated antibody (#042-206, ProteinSimple®). In probe 2, Mfn2 and GAPDH primary antibodies were multiplexed and both were decorated with ready to use goat anti-mouse secondary HRP-conjugated antibody (#042-205, ProteinSimple®). For preparing the final primary antibody dilution mix, milk-free antibody diluent buffer 2 was used (#042-203, Bio-Techne). Signal was developed by using enhanced chemiluminescence (ECL) reagents provided in the secondary antibody module (#DM-001, ProteinSimple®) and according to the manufacturer´s instructions. Data were analyzed using Compass for Simple Western software. Images from the high dynamic range 4.0 were utilized for the analysis, with peaks automatically detected and quantified (both peak height and area were examined). Values of Miro1, Mfn2 and VDAC peaks were normalized to GAPDH peak value.

Recognizing that some antibodies incompatible with the capillary system might still be effective in traditional Western blotting, we aimed to verify that antibodies against Miro1 from different companies bind the same target protein. To this end, we conducted a preclearing assay in which Miro1 protein was immunoprecipitated from fibroblast lysate using various Miro1 antibodies. This was followed by addition of IgG Sepharose beads to separate the precleared lysate from the immunoprecipitated Miro1 protein. Two controls were included to test validity of the assay. The first was lysate with no antibody, serving as the input lysate for comparison. The second was a precleared lysate using an antibody against Mfn2. The precleared lysate, depleted of either Miro1 or Mfn2, was then analyzed using the capillary blotting system and probed with Miro1 antibodies (#HPA010687 and #77C5), Mfn2 and GAPDH, which served as loading controls; as shown in Fig. S4B. A consistent reduction in the Miro1 band was detected by #HPA010687 (right panel) and #77C5 (left panel) across precleared lysates using the antibodies: #HPA01687, #NBP1-89011, and, to lesser extent, #PA5-42646. This validates the reliability and specificity of these antibodies in recognizing Miro1 in fibroblast lysates. In contrast, lysate precleared with the antibody #WH0055288M1 did not show a reduced Miro1 signal, suggesting that this antibody does not efficiently bind Miro1 protein in our hands.

Next, we aimed to optimize the dilution of the Miro1 antibody to ensure it is used at saturating concentrations, allowing any observed changes in signal to be attributed solely to changes in protein amount. Achieving the optimal dilution is crucial for quantitative analysis, enabling precise comparison of protein levels both within the same sample under different treatment conditions and across multiple samples. To determine the optimal dilution, we conducted an antibody titration assay using Miro1 (#HPA010687), which was serially diluted in a range from 1:5 to 1:60, as shown in Fig. S4C. The chemiluminescent signal of Miro1 was quantified and plotted as peak area (band intensity) against antibody dilution (Fig. S4D). The antibody began to saturate at a dilution of approximately 1:10, which was selected as the optimal dilution for subsequent analyses.

To further validate the specificity of the Miro1 signal, we analyzed Miro1 knockdown fibroblast lysates from two batches with transfection times of 7 and 10 days. As depicted in Fig. S4 E, F, we observed a significant reduction in Miro1 signal in all knockdown cell lines using #HPA01687 antibody, compared to the control, confirming the antibody’s specificity. GAPDH was used as a loading control, and Mfn2 served as a MOM substrate control. In line with previous study^28^, we observed elevated levels of Mfn2 in the knockdown cells (Fig. S4E), reflecting the disruption of mitochondrial homeostasis due to Miro1 knockdown. The Area under the curve representing the chemiluminescence intensity of Miro1 in all cell lines is shown in Fig. S4F.

## Lysate preclearing assay

The fibroblast (cell line ID16860, Tübingen Biobank) was lysed from T75 flasks with ice-cold lysis buffer (100 mM Tris, 150 mM NaCl, 1 mM EGTA, 1 mM EDTA, 1% Triton X-100, 0.5% sodium deoxycholate, 1 mM PMSF, 1:10 protease inhibitor cocktail (539134, Calbiochem, San Diego, United States of America)). The lysate was diluted to final concentration of 1 mg/mL prior to immunoprecipitation. Miro1 antibodies from various suppliers (Thermofisher, Sigma/Merck, Novus) were added to 100 µL of fibroblast lysate at a 1:10 dilution (10 µL antibody per 100 µL lysate). The mixtures, including no antibody control and Mfn2 antibody control, were incubated overnight on a rotator at 4 °C to ensure antibody binding to the target protein. Magnetic Protein G agarose beads (Thermofisher #88848) were washed three times with PBS, and 10 µL of beads per 100 µL of lysate were added to the antibody-lysate mixtures. The samples were incubated for an additional 2 h. at 4 °C to allow binding of the beads to the antibody-protein complexes. After incubation, the beads together with the bound material were separated from the lysate by magnetic stand. The supernatant (cleared lysate) was carefully transferred to a new tube. The precleared lysate was then analyzed by automated Western blotting JESS Simple Western™ (Bio-Techne) using the following Miro1 antibodies for detection: Miro1_77C5 (AcureX Biosciences) and Miro1_HPA01687 (Sigma).

## Miro1 knockdown in fibroblast cell lines

Miro1 knockdown in healthy fibroblast cell lines was achieved using two lentiviruses designed with shRNA target sequences (shMIRO1#653: GATATCTCAGAATCGGAATTT; shMIRO1#761: ATGATCCTTTGGGTTCTATAA) to silence protein expression. A third lentivirus, containing shRNA sequence with no target sequence, served as the control. Fibroblast cell line (ID18075, Table 1 provided by Tübingen Neuro-Biobank) was cultured until 70% confluency. The standard growth medium was replaced with a medium containing the lentiviruses, and cells were incubated at 37 °C with 5% CO2 for 8 h. Following incubation, the virus-containing medium was removed and replaced with fresh fibroblast medium (high glucose DMEM supplemented with 10% fetal bovine serum and 1% penicillin-streptomycin). Protein extraction from fibroblasts was performed 7- and 10-days post-infection.

## Data analysis, machine learning and statistics

Quantification of Miro1, Mfn2, and VDAC protein levels was performed using Chemiluminescence in the Simple Western instrument software Compass for SW. This software is available online at: https://www.bio-techne.com/resources/instrument-software-download-center?filters%5Binstrument_category%5D%5B0%5D=372.

Peaks corresponding to Miro1, Mfn2, and VDAC, were identified and named in the software. For each capillary to be analyzed, Peak Fit (Threshold and Width) was optimized and adjusted as needed to ensure the green area under the target peak aligns with the curve’s black border. Protein peaks were normalized to GAPDH peak (loading control), ensuring equal loading across samples. After automatic normalization, the corrected values of proteins were used to compare levels between samples.

For machine learning predictions, we employed several available models from the caret R package including random forest (rf), k-nearest neighbors (knn), bayesian generalized linear model (bayes), linear discriminant analysis (lda), and boosted generalized linear model (glm)^29^. In order to evaluate model performance, we used receiver operating characteristic (ROC) curve analyses using the MLeval R package (https://github.com/crj32/MLeval). Performance assessment was done using 10-fold cross-validation. Area under the curve (AUC) or ROC curves was used as indicator for model performance.

GraphPad Prism (version 10.4.1) was used to create the graphs and perform statistical analysis. The results are shown as mean quantification value across independent experiments ± standard deviation (SD). Distribution of the data was assessed in Prism and in all cases the data was not normally distributed or n was too low. Non-parametric statistical tests were used to compare groups and are stated in the figure legends. Simple linear regression and Pearson correlation coefficients were calculated in Prism and all information is shown on the appropriate graphs. All *P* values <0.05 are stated numerically on each graph.

## Polygenic risk scores

Polygenic risk scores (PRSs) were calculated and taken for different gene-sets for the Courage-PD cohort (7270 iPD cases and 6,819 healthy controls) as previously described in Arena et al^23^. In short, genome-wide PD-PRSs were calculated using the PRSice2 R package^30^ with default settings. PRSs for the pathway-specific gene sets were generated using the PRSet function in PRSice2, using only risk alleles within gene regions outlined in the different gene lists. Mitochondrial gene-sets and lysosomal gene-sets were obtained from the literature or the Molecular Signatures Database (MsigDB) v7.5.1. For eachPRS the 10/20/80/90% percentiles were calculated for the whole Courage-PD cohort. The three samples included in this study were then classified as very high (90), high(80), low(20), and very low (10) risk.

## Supplementary information

Supplementary figures legends

Supplementary Table

Supplementary figures 1 and 2

## Data Availability

Raw data is available as Supplementary Table 1 and any other anonymized clinical data is available upon on reasonable request. Full, detailed protocols can be accessed at protocols.io https://www.protocols.io/view/optimized-automated-capillary-western-blotting-met-e6nvwe5z9vmk/v1. All raw data and lists to be deposited at Mendeley data, https://data.mendeley.com/ (to be updated with full link).
