# Supplementary material for: Methodological validation of Miro1 retention as a candidate Parkinson’s disease biomarker
Source: NPJ Parkinsons Dis. 2025 Sep 15;11:270. doi: 10.1038/s41531-025-01115-8 (PMC12436598; doi:10.1038/s41531-025-01115-8)
Supplement: Supplementary file 1 — Supplementary figures legends [file 41531_2025_1115_MOESM1_ESM.pdf]

Supplementary Figures

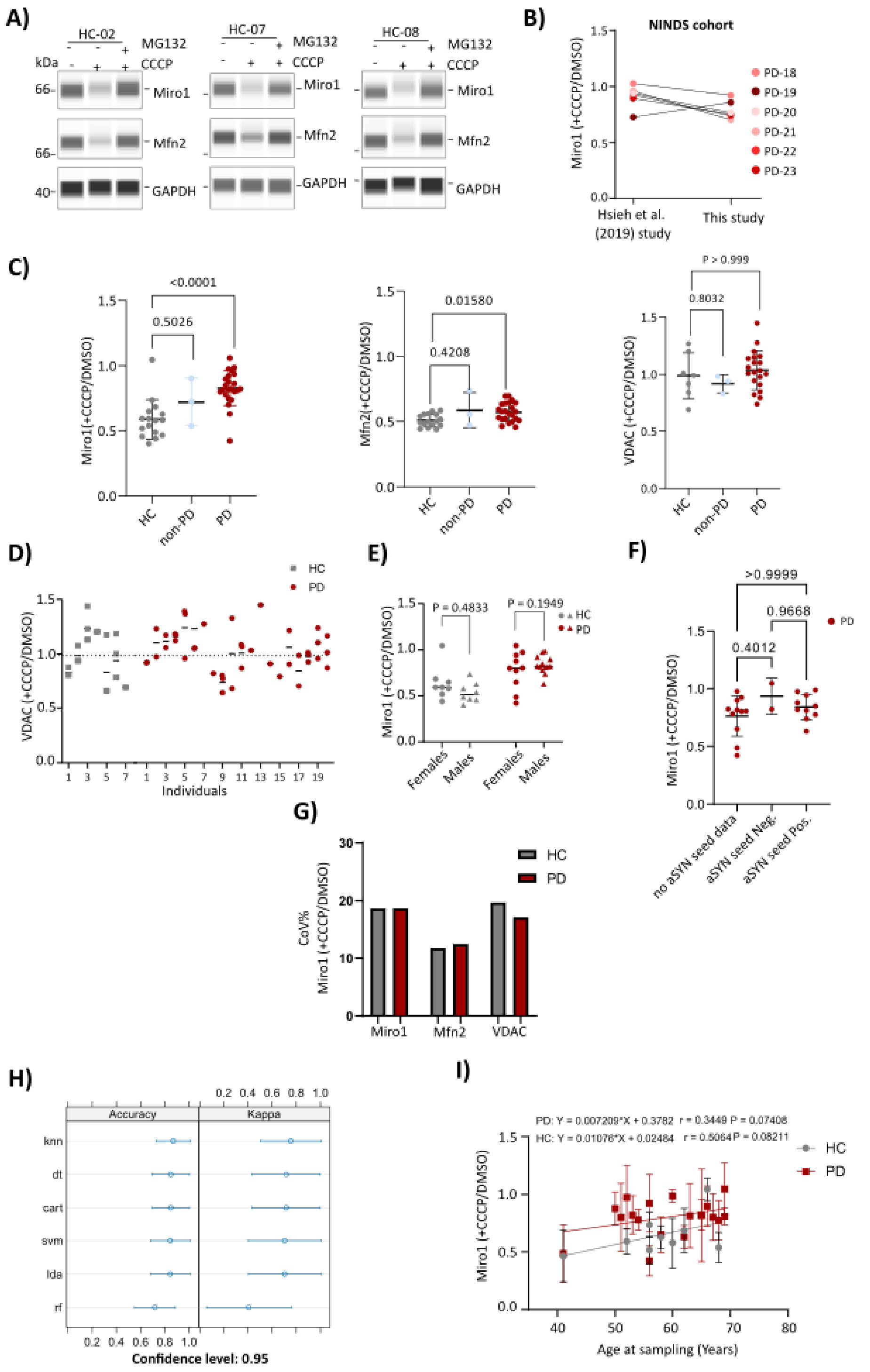

Figure S1: (A) Fibroblast cell lines from three healthy controls were treated with DMSO (vehicle control), 40 µM CCCP (to induce depolarization), and CCCP combined with 10 µM of the proteasome inhibitor MG132. **(B)** A line graph compares Miro1 retention levels (+CCCP/DMSO) in the same NINDS fibroblast cell lines (PD18-PD23) analyzed in this study and the Hsieh et al. (2019) study. **(C)** Scatter-plot graphs display the distribution of protein (Miro1, Mfn2 and VDAC) depolarization-response (degradation levels) across individuals in HC (gray), non-PD

(blue), and PD (red) groups**,** one-way ANOVA of multiple comparisons using Kruskal-Wallis test was used to assess the statistical significance between HC and non-PD groups and HC and PD groups with P-values stated numerically on the graph. **(D)** Scatter-plot graph display the distribution and average depolarization-response value of VDAC across individuals in HC and PD groups. **(E)** Distribution of average Miro1 depolarization response across individuals in the HC (gray) and PD (red) groups is compared between females and males. The statistical significance between females and males in the HC and PD groups was assesses using Mann-Whitney test with P-values stated numerically on the graph. **(F)** Miro1 depolarization-response distribution among PD patients is compared across the following categories: positive α-synuclein data, negative α-synuclein data and no α-synuclein data. One-way ANOVA of multiple comparisons using Kruskal-Wallis test was used to assess the statistical significance between the groups with P-values stated numerically on the graph. **(G)** Graphs represent the coefficient variance (%CoV) of the depolarization-response for the proteins (Miro1, Mfn2 and VDAC) in HC and PD groups. **(H)** Accuracy and Cohen's Kappa statistics for different models, including Random Forest (rf), Linear Discriminant Analysis (lda), Support Vector Machine (svm), Classification and Regression Trees (cart), Decision Tree (dt), and K-Nearest Neighbors (knn). Higher accuracy and Kappa values indicate better model performance. Error bars represent **95% confidence intervals.** **(I)** Correlation between Miro1 average retention levels ± SD, and age at sampling (AAS) across age-matched individuals from HC and PD cohorts. Pearson correlation (r) and Linear regression analysis were performed.

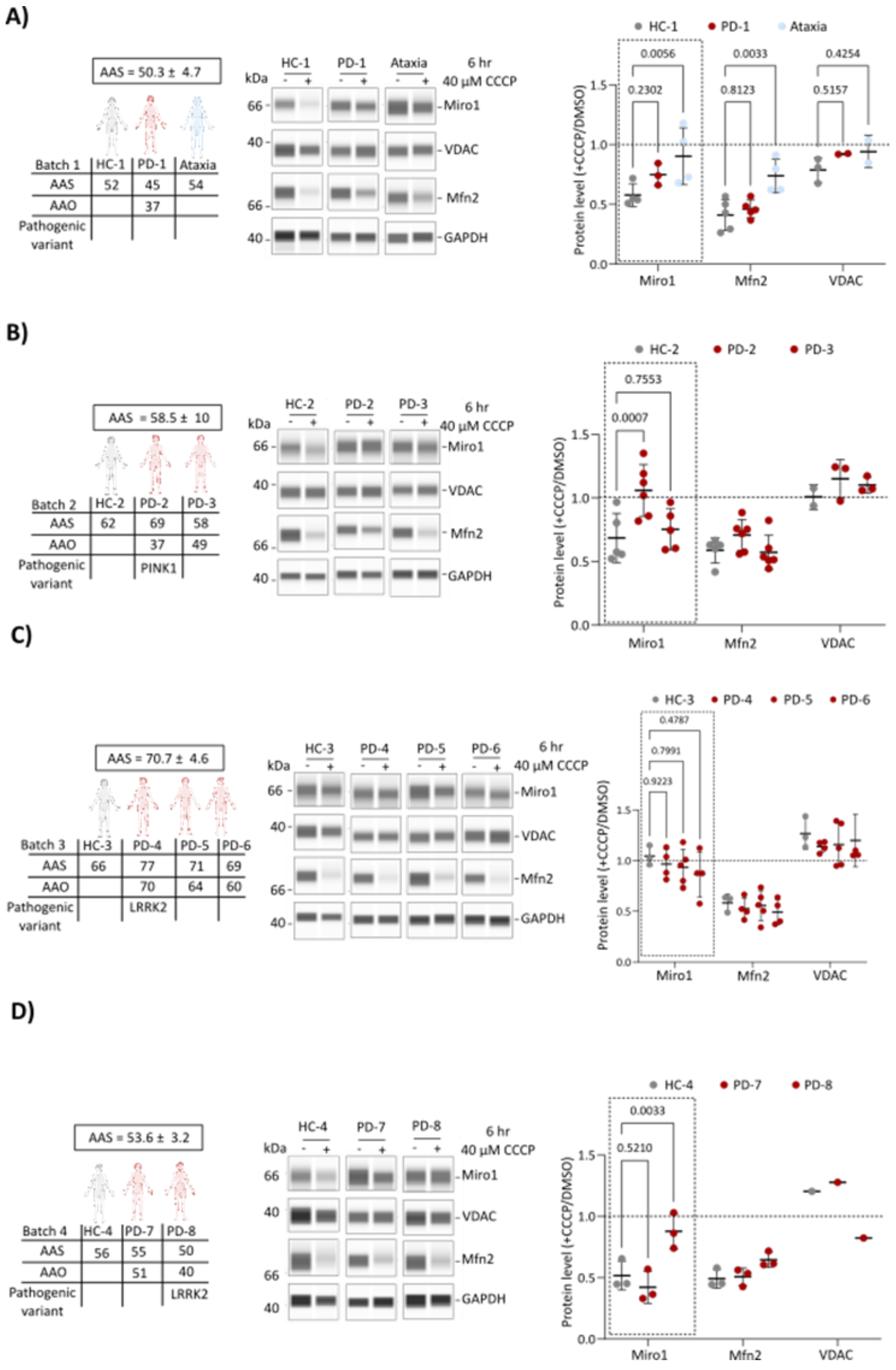

Figure S2: Miro1 response to mitochondrial depolarization in an independent batches-females: (A-D) Whole-cell lysates from fibroblasts derived from female healthy individuals and Parkinson's disease (PD) patients (Tübingen cohort) were analyzed using simple western blotting (JESS, Bio-Techne). Fibroblast cell lines were grouped based on age- and sex- matched criteria, with each group consisting of one healthy control (gray), 2-3 PD patients (red), and one non-PD control (light blue). The mean age at sampling (AAS) ± SD for each group is indicated. For each experimental group, fibroblasts were seeded and treated simultaneously with either DMSO (vehicle control, -) or 40 μM CCCP for 6 hours to induce mitochondrial depolarization. Miro1 protein levels were assessed under depolarizing conditions alongside control proteins, including mitochondrial markers (Mfn2 and

VDAC) and the cytosolic housekeeping protein GAPDH (loading control). Protein intensities were quantified using CompassForSW software and normalized to GAPDH within the same lane. The response to depolarization was calculated as the ratio of protein levels in CCCP-treated samples to vehicle control (+CCCP/DMSO). Scatter-plot graphs display the distribution and average depolarization response values for each protein across individuals, based on at least three biological replicates. Error bars represent the standard deviation (SD) for each individual.

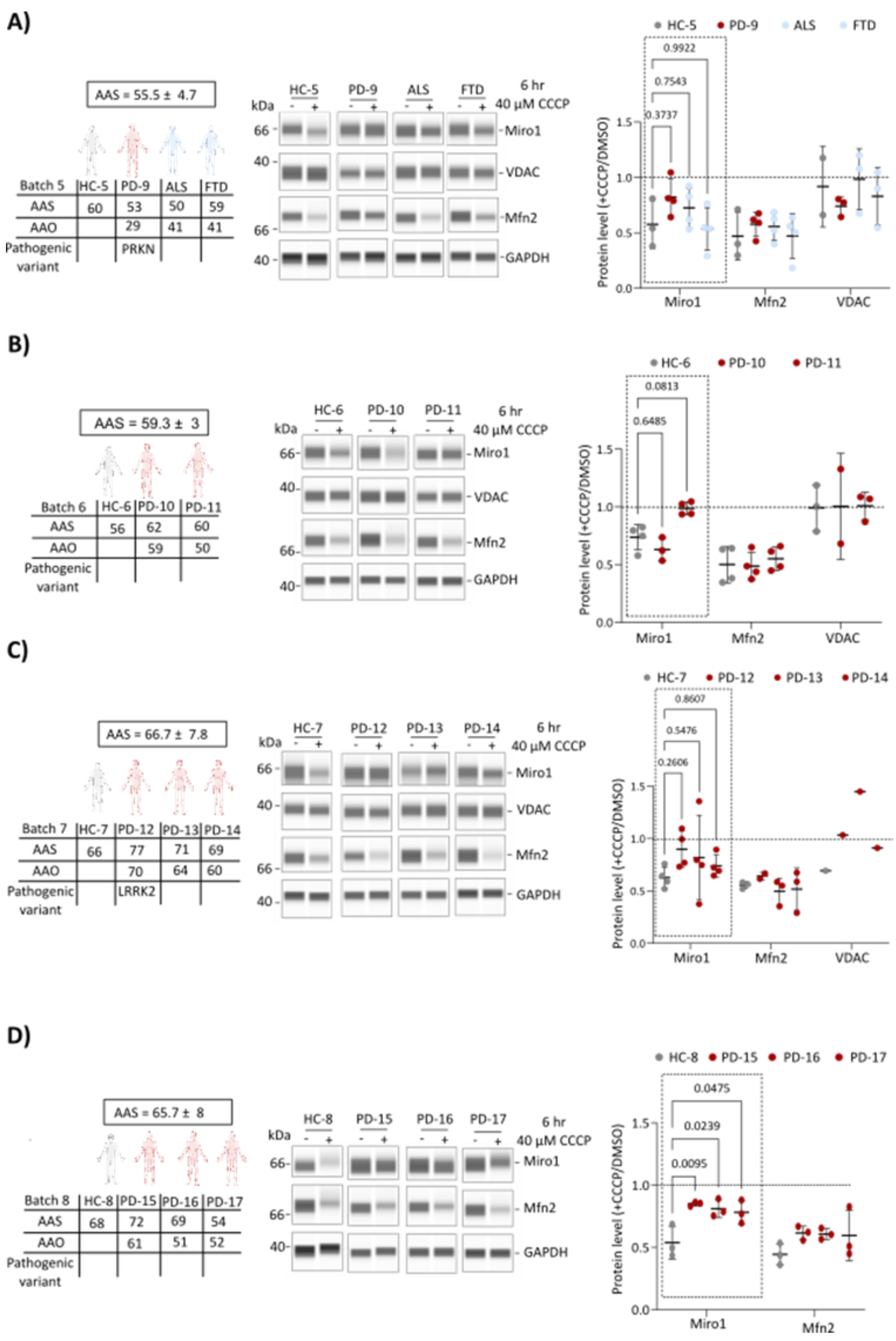

Figure S3: Miro1 response to mitochondrial depolarization in independent batches-males (A-D) Whole-cell lysates from fibroblasts derived from male healthy individuals and Parkinson's disease (PD) patients (Tübingen cohort) were analyzed using simple western blotting (JESS, Bio-Techne). Fibroblast cell lines were grouped based on age- and sex- matched criteria, with each group consisting of one healthy control (gray), 2-3 PD patients (red), and two non-PD control (light blue). The mean age at sampling (AAS) ± SD for each group is indicated. For each experimental group, fibroblasts were seeded and treated simultaneously with either DMSO (vehicle control, -) or 40 µM CCCP for 6 hours to induce mitochondrial depolarization. Miro1 protein levels were assessed under depolarizing conditions alongside control proteins, including mitochondrial markers (Mfn2 and VDAC) and the cytosolic housekeeping protein GAPDH (loading control). Protein intensities were quantified using CompassForSW software and normalized to GAPDH within the same lane. The response to depolarization was calculated as the ratio of protein levels in CCCP-treated samples to vehicle control (+CCCP/DMSO). Scatter plot graphs display the distribution and average depolarization response values for each protein across individuals, based on at least three biological replicates. Error bars represent the standard deviation (SD) for each individual.

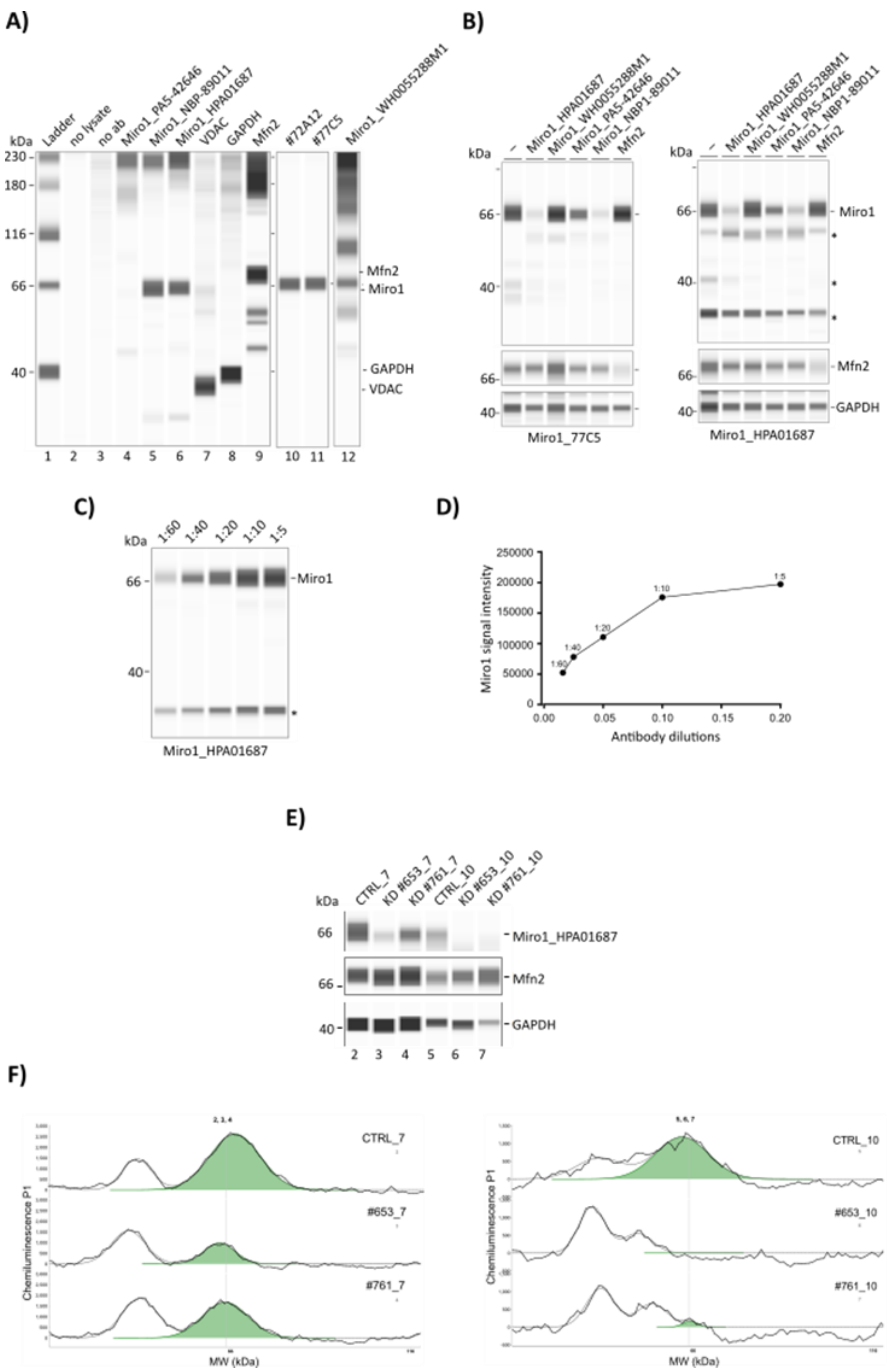

Figure S4: Confirmation of Miro1 antibody specificity: **(A)** Multiple lanes of the same fibroblast lysate were used to test the compatibility and signal intensity of different primary antibodies with the capillary system. Each lane was blotted with a primary antibody against: Mfn2, VDAC, GAPDH, or Miro1 from different companies (Thermo Fisher #PA5-42646, Novus #NBP-89011, Sigma #HPA01687 and Sigma WH0055288M1). Two controls were included: no lysate control (lane 2) and no antibody control (lane 3). **(B)** Fibroblast lysates precleared with different Miro1 antibodies were analyzed using the capillary Western blot system. All lanes were decorated with

antibodies against Mfn2, GAPDH, and Miro1 (#HPA01687). Bands marked with an asterisk indicate non-specific binding. **(C)** Different dilutions (1:5, 1:10, 1:20, 1:40, and 1:60) of the Miro1 antibody (#HPA01687) were tested on the same lysate concentration (0.8 mg/mL) to determine the optimal dilution. **(D)** Intensity values of Miro1 bands at different dilutions were plotted to determine the optimal antibody concentration. **(E)** Lysates from knockdown (KD) fibroblast cell lines (#653 and #761) and their corresponding controls (CTRL) were analyzed using the capillary system and decorated with the indicated antibodies. **(F)** Miro1 bands intensity from control and KD lysates are represented by the green area under the peak.
