# Supplementary material for: Methodological validation of Miro1 retention as a candidate Parkinson’s disease biomarker
Source: NPJ Parkinsons Dis. 2025 Sep 15;11:270. doi: 10.1038/s41531-025-01115-8 (PMC12436598; doi:10.1038/s41531-025-01115-8)

Figure 1A – Healthy males

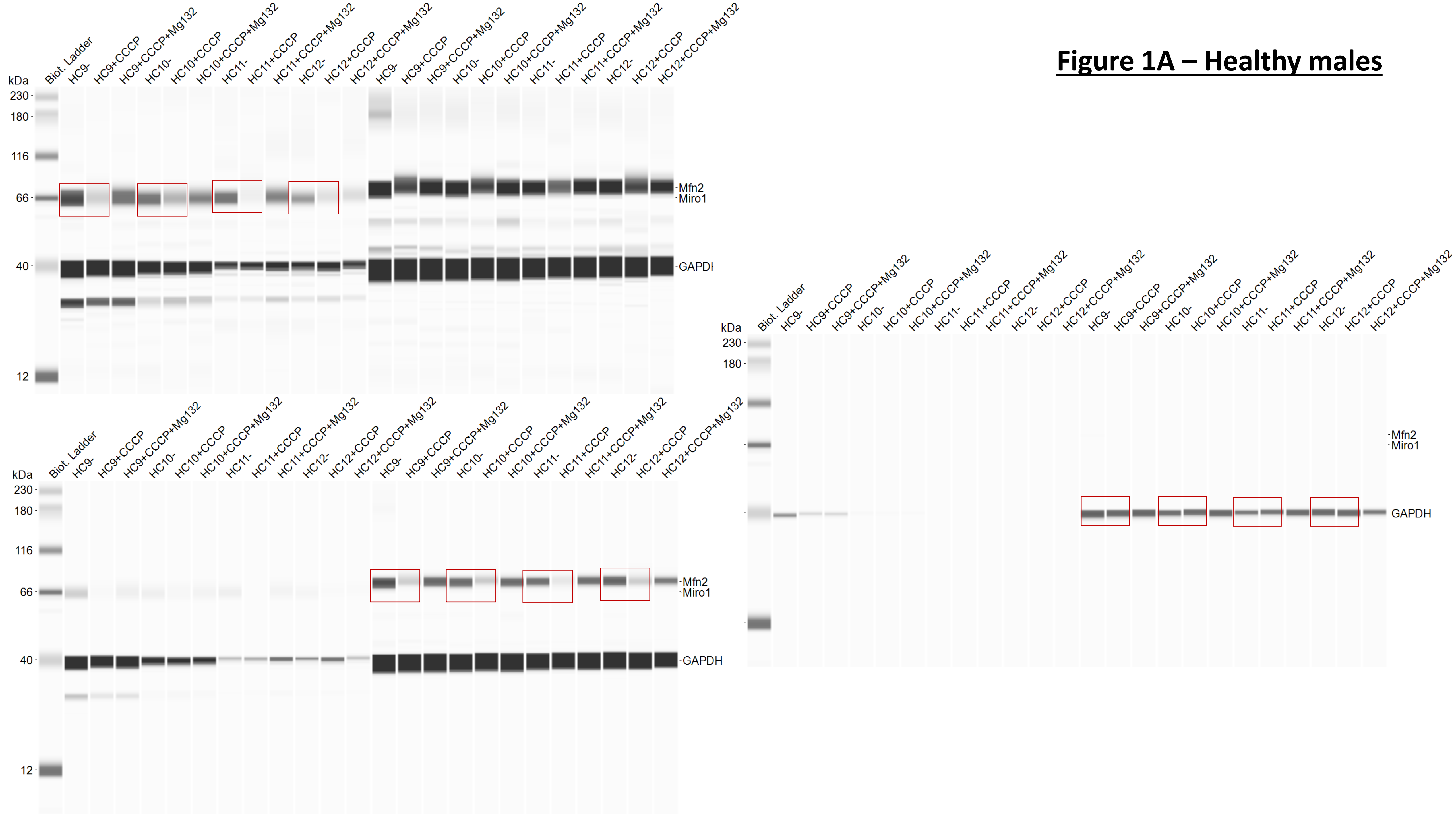

Figure 1A – Healthy females

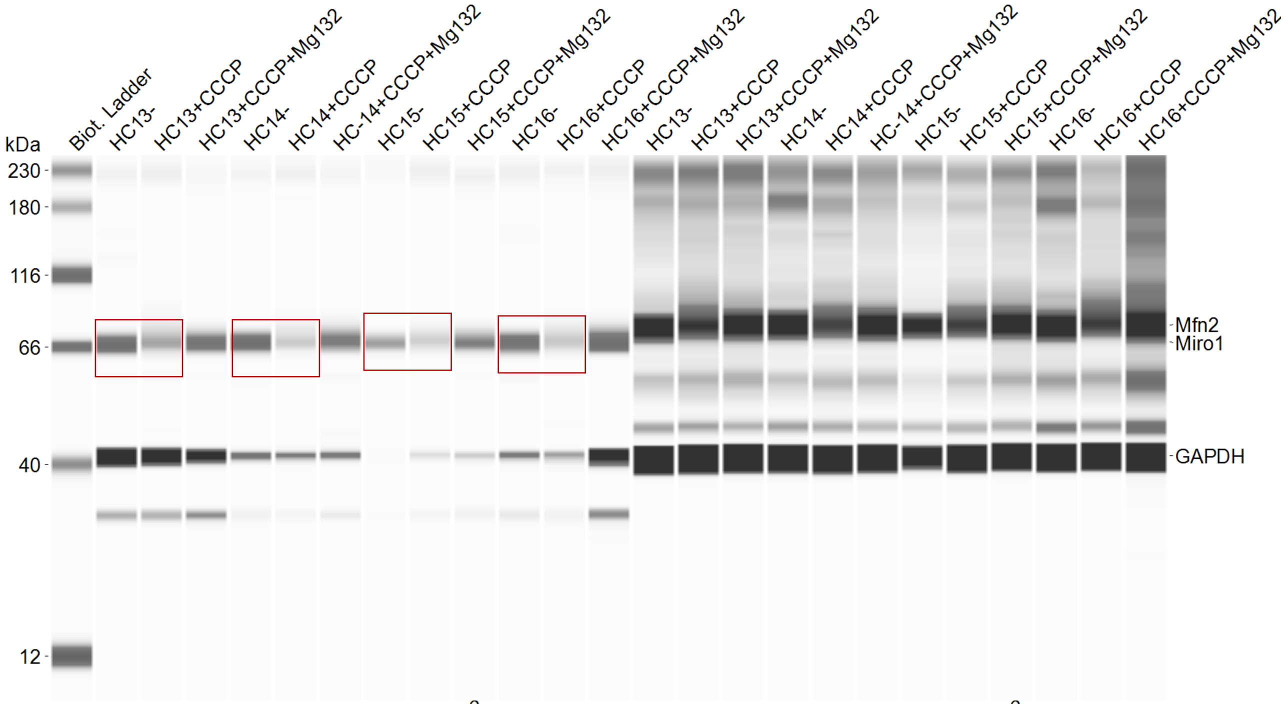

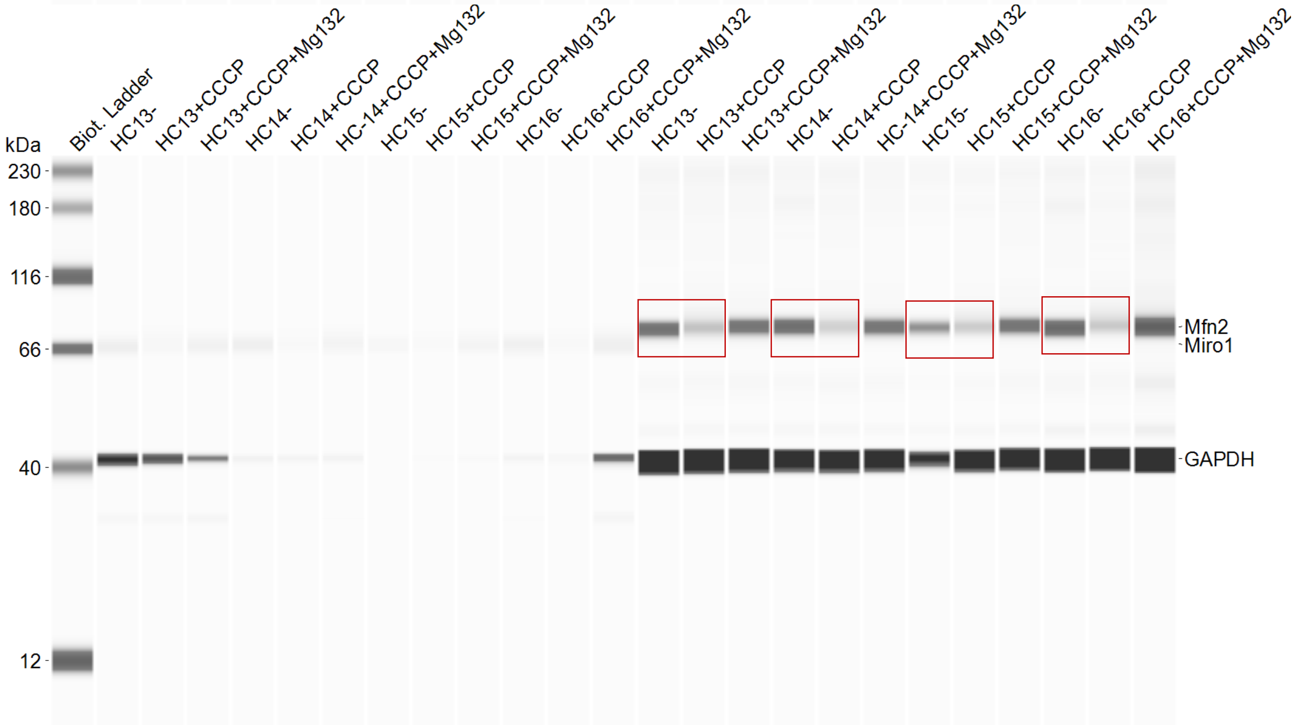

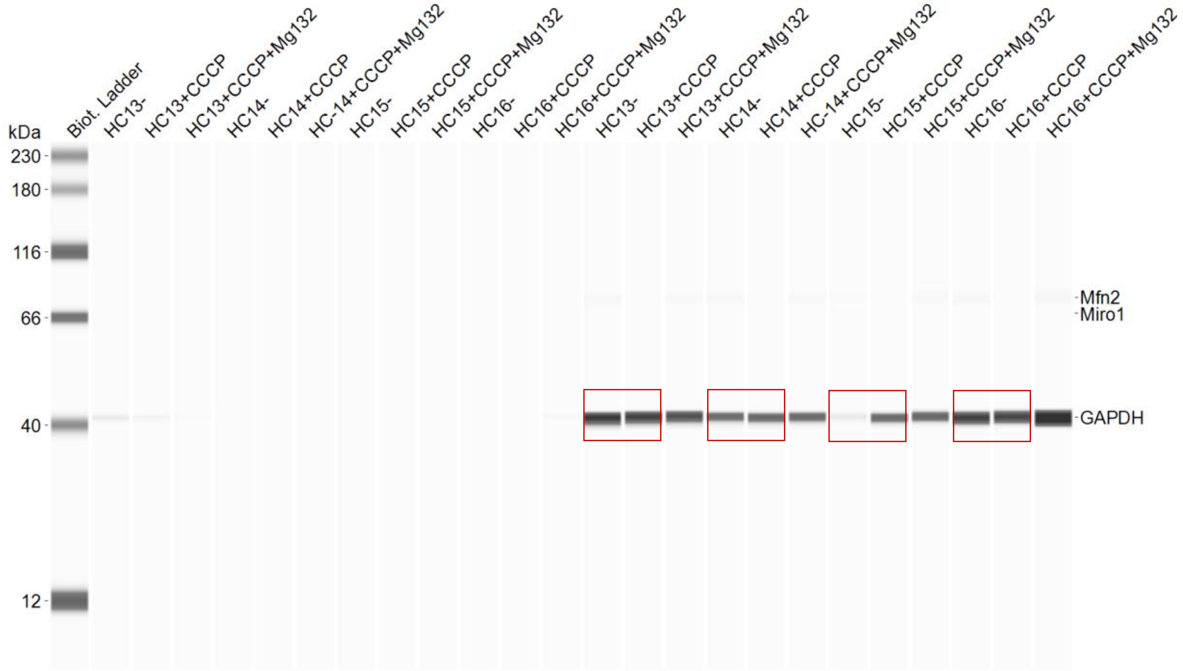

Figure 1B – NINDS

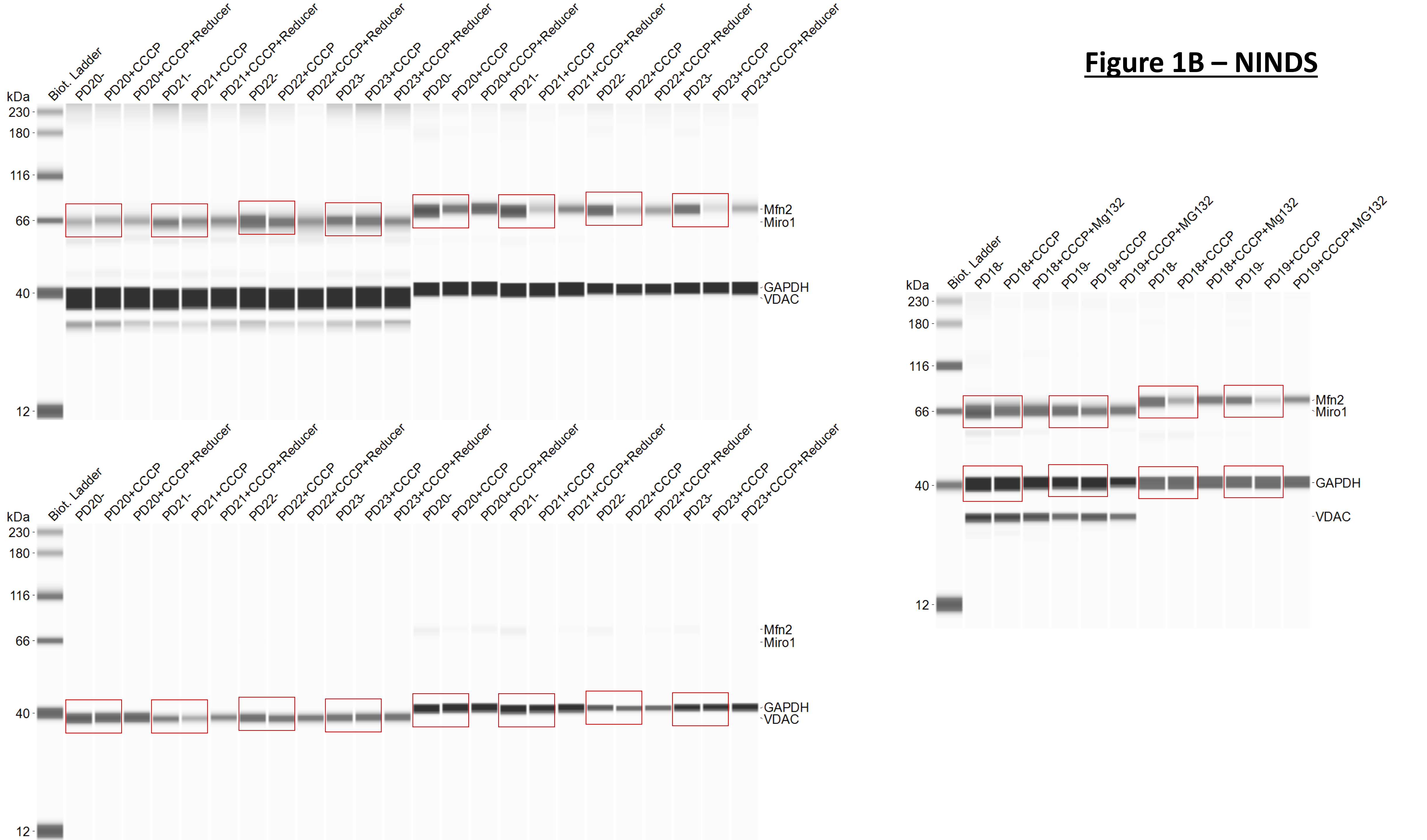

Figure 1C – Tübingen cohort HC1, HC2, HC5, HC6

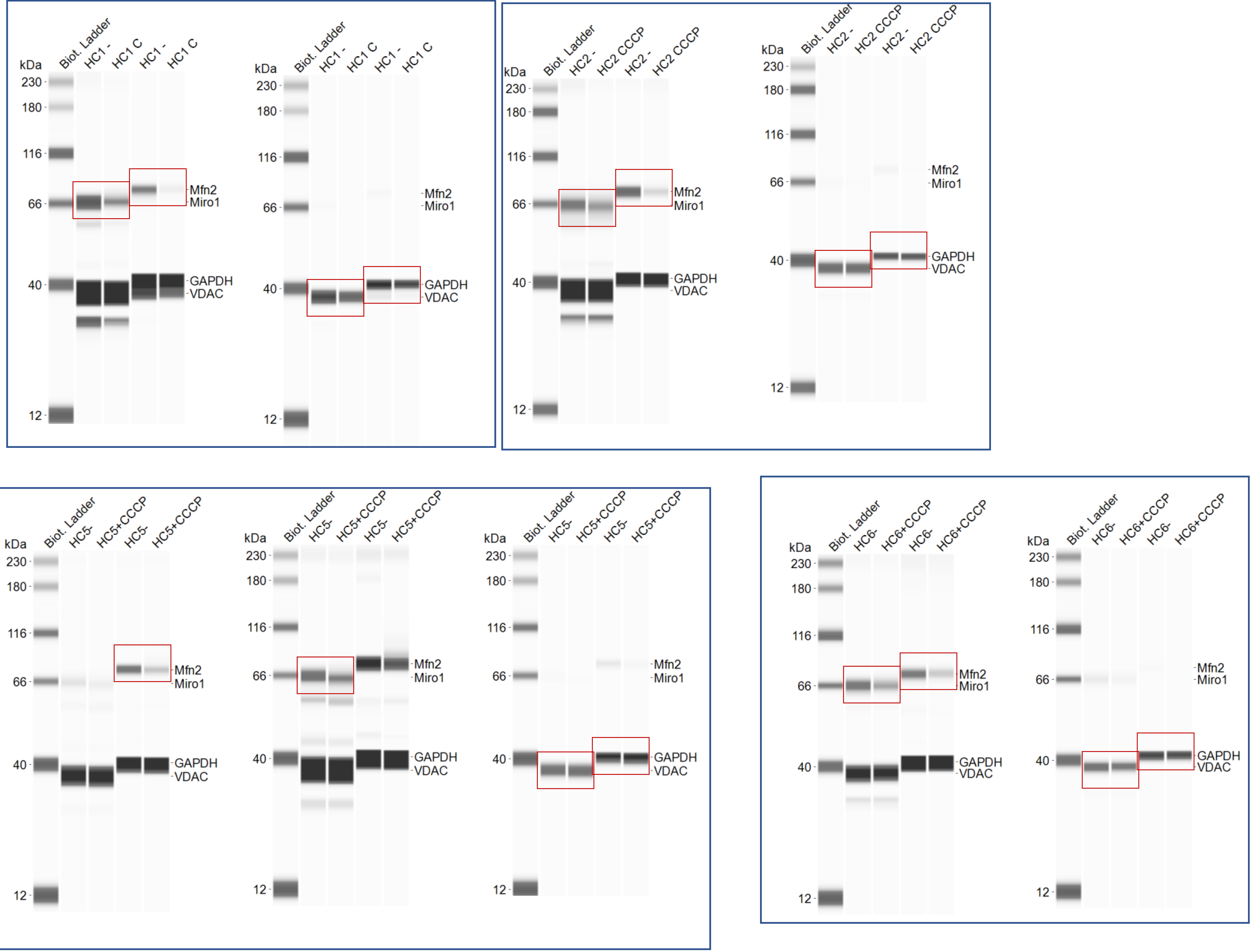

Figure 1C – Tübingen cohort PD1, PD2, PD9, PD11

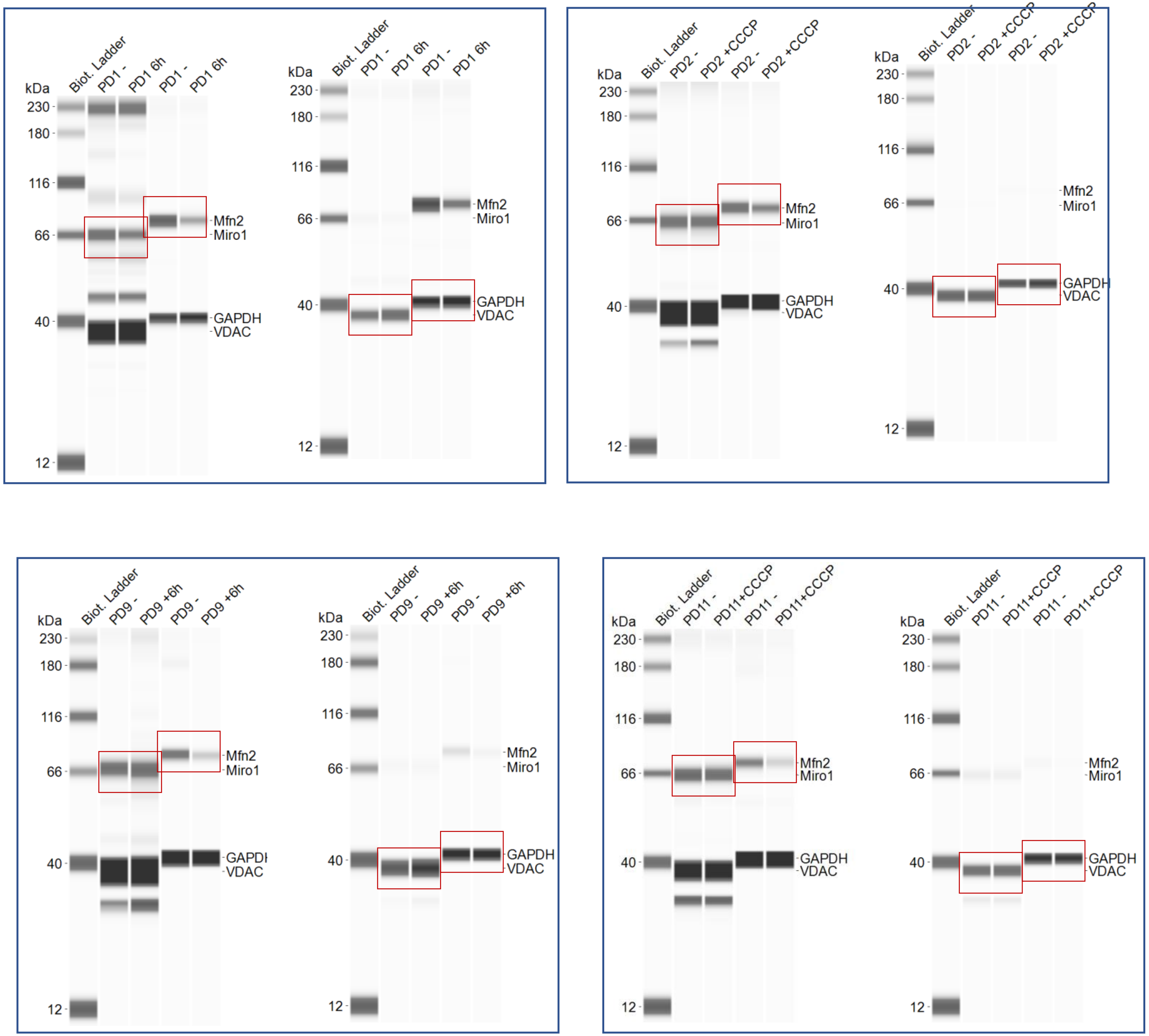

Figure 2A – HC1

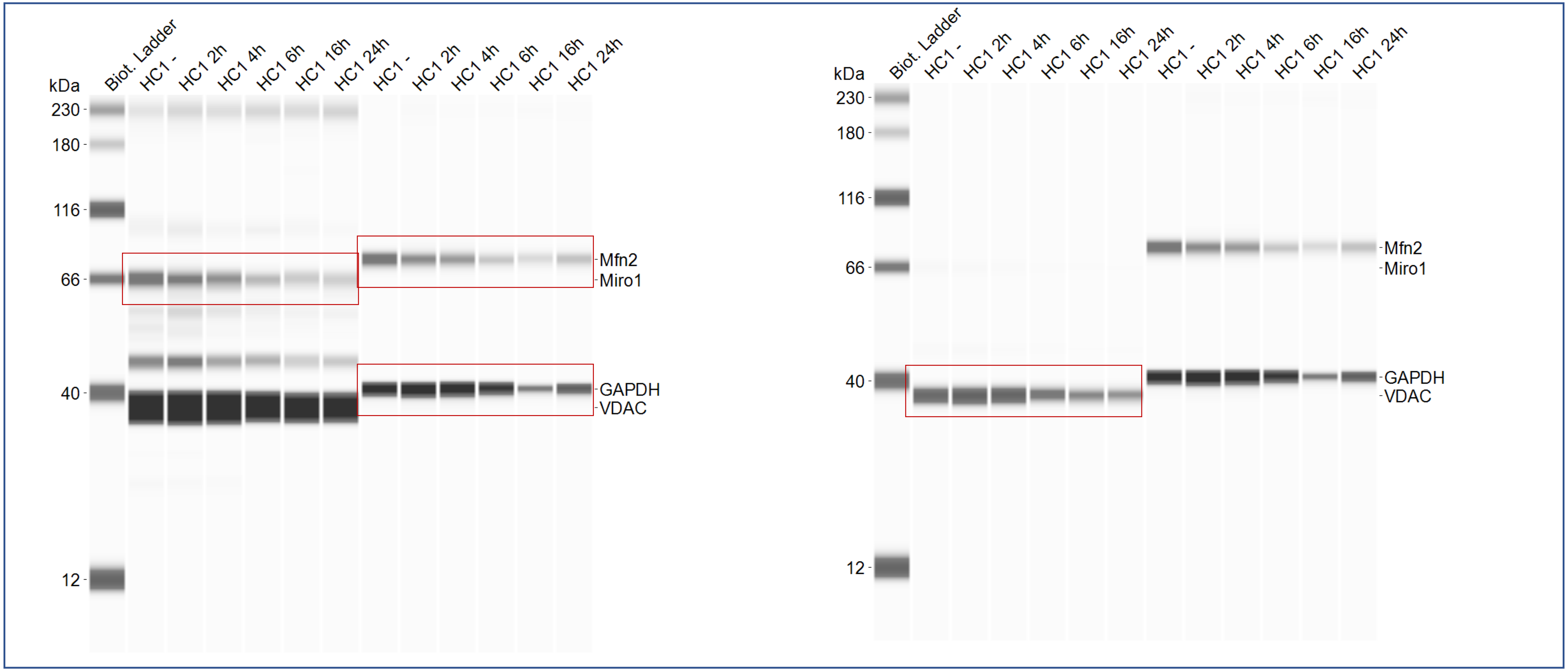

Figure 2A – HC9, PD9

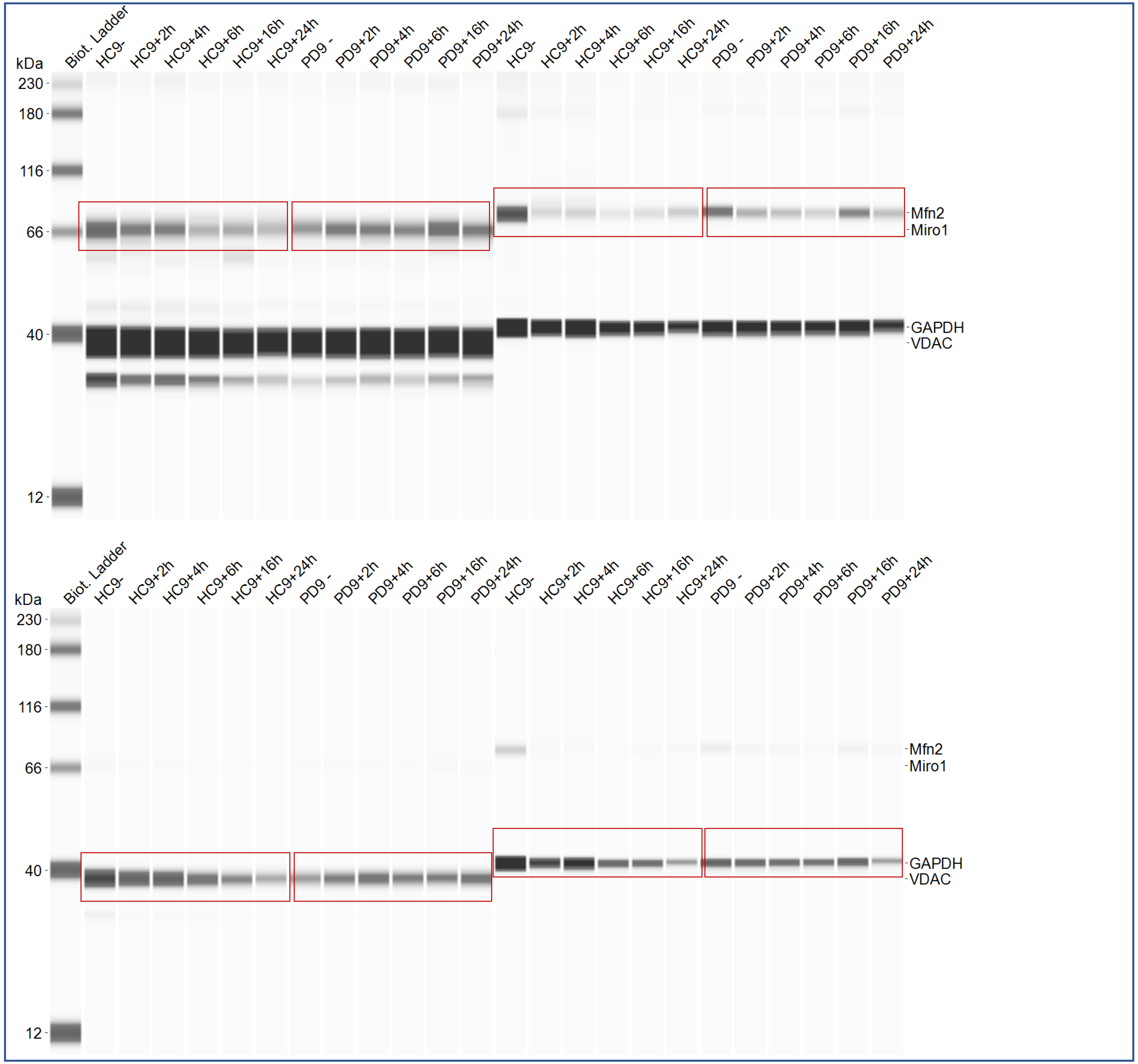

Figure 2A – PD1, PD2

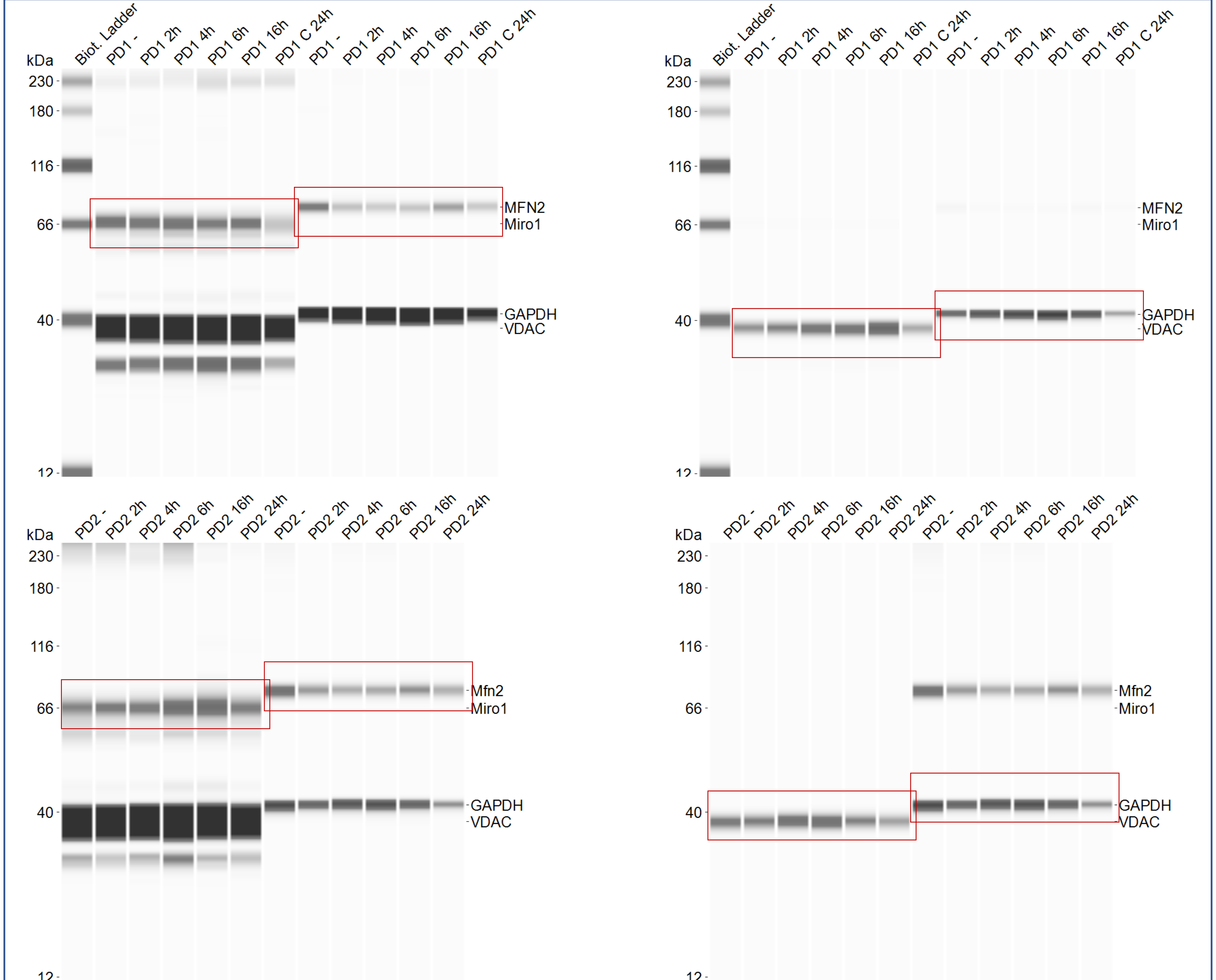

Figure 2A – PD5, PD6

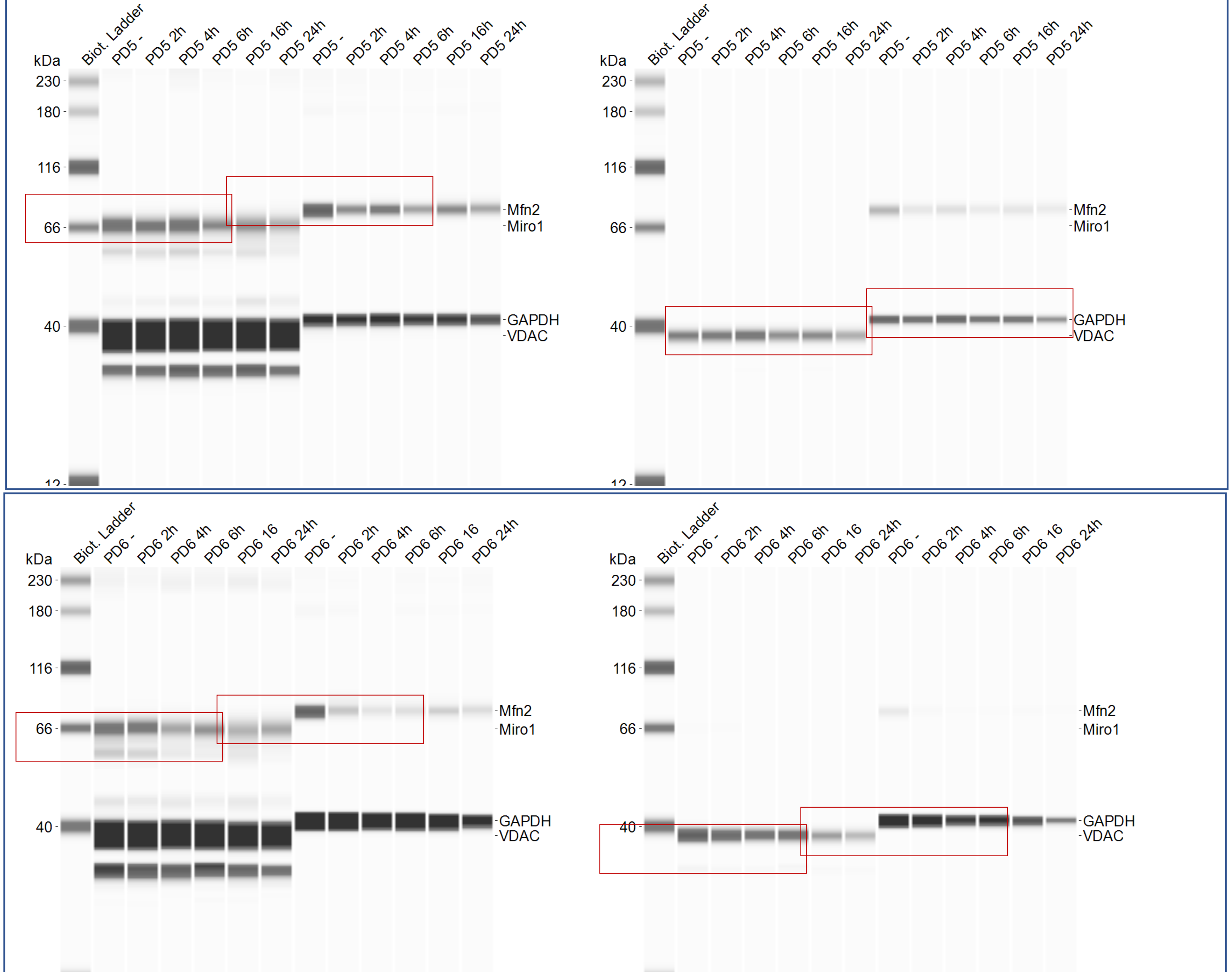

Figure 4C

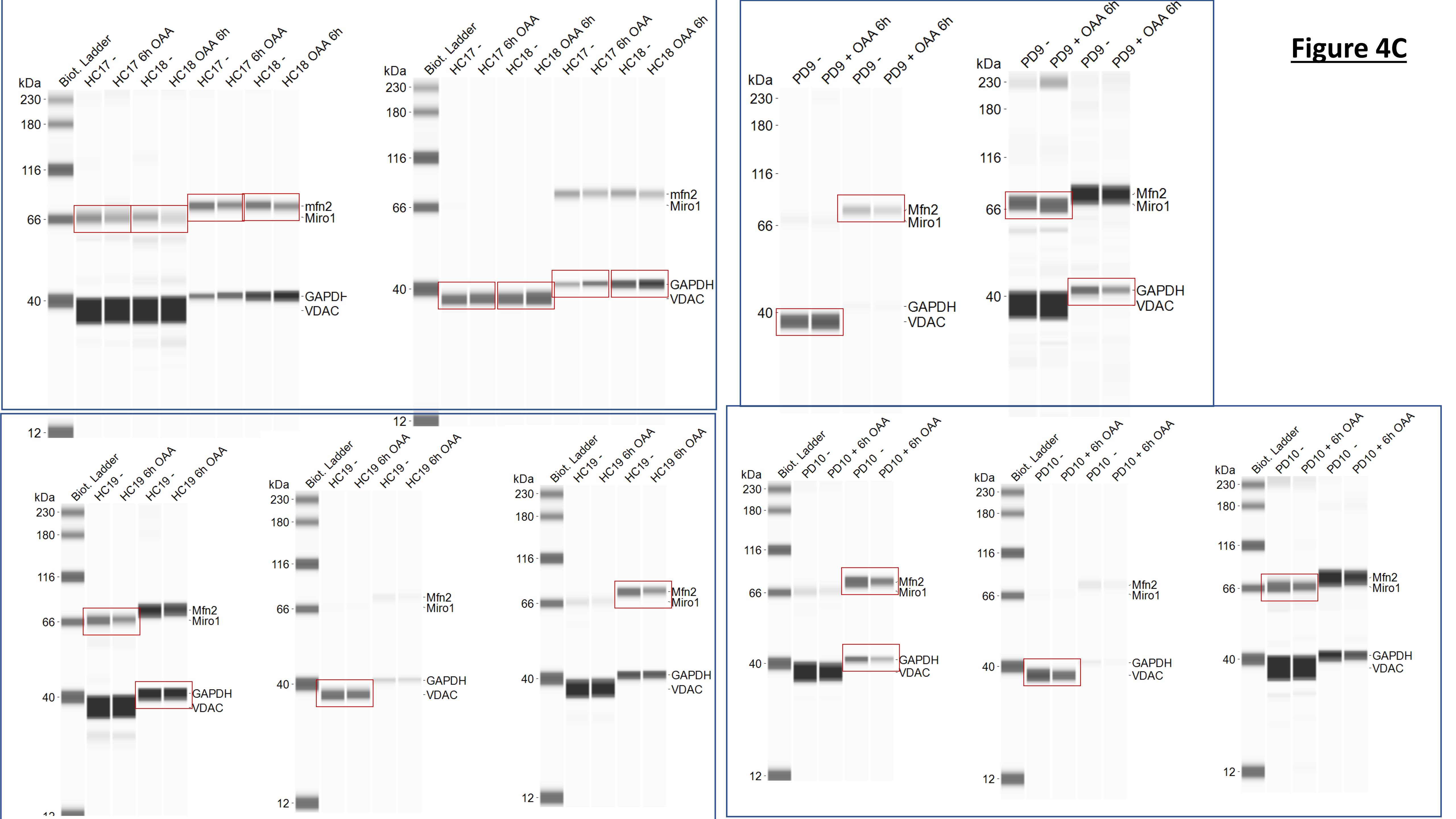

kDa
Biot. Ladder
HC17 -
HC17 6h OAA
HC18 -
HC18 OAA 6h
HC19 -
HC19 6h OAA
PD9 -
PD9 + OAA 6h
PD10 -
PD10 + 6h OAA
230
180
116
66
40
12
mfn2
Miro1
Mfn2
GAPDH
VDAC

Figure 4C

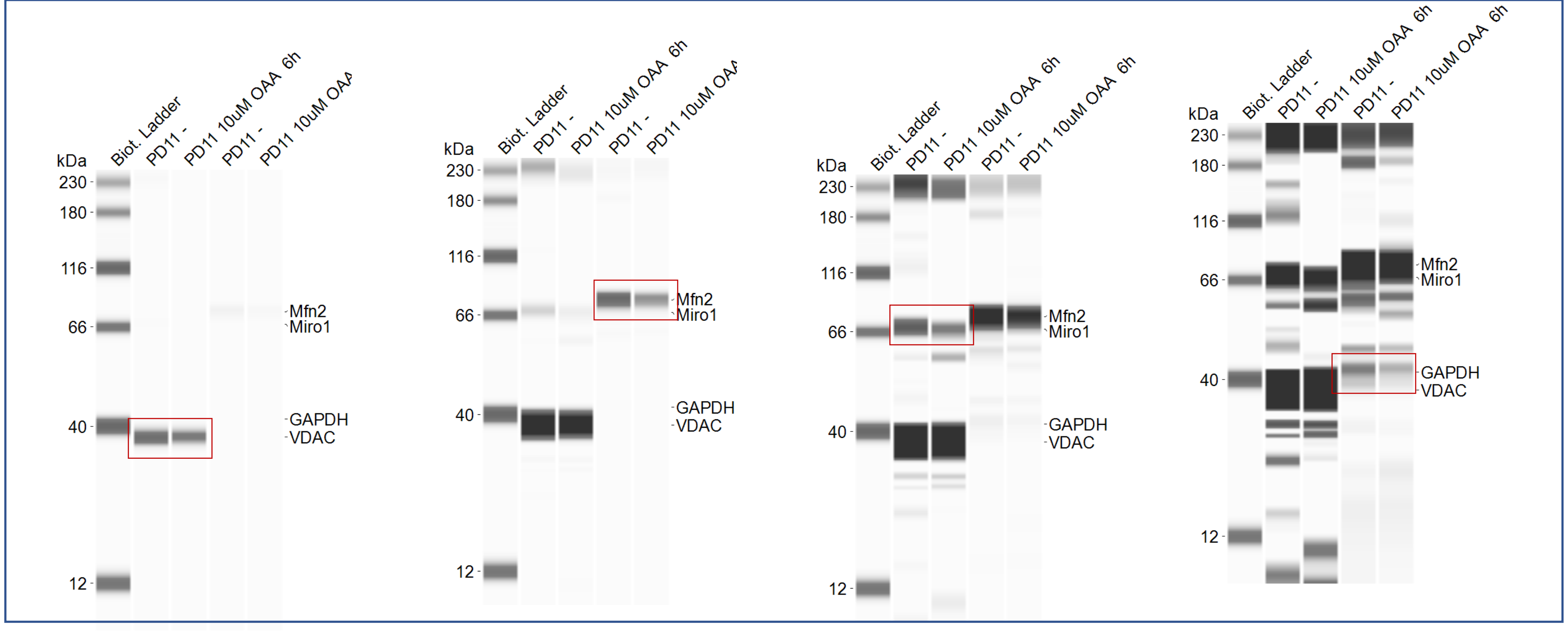
kDa
Biot. Ladder
PD11 -
PD11 10uM OAA 6h
PD11 -
PD11 10uM OAA
230
180
116
66
40
12
Mfn2
Miro1
GAPDH
VDAC
PD11 10uM OAA 6h

Figure 4E

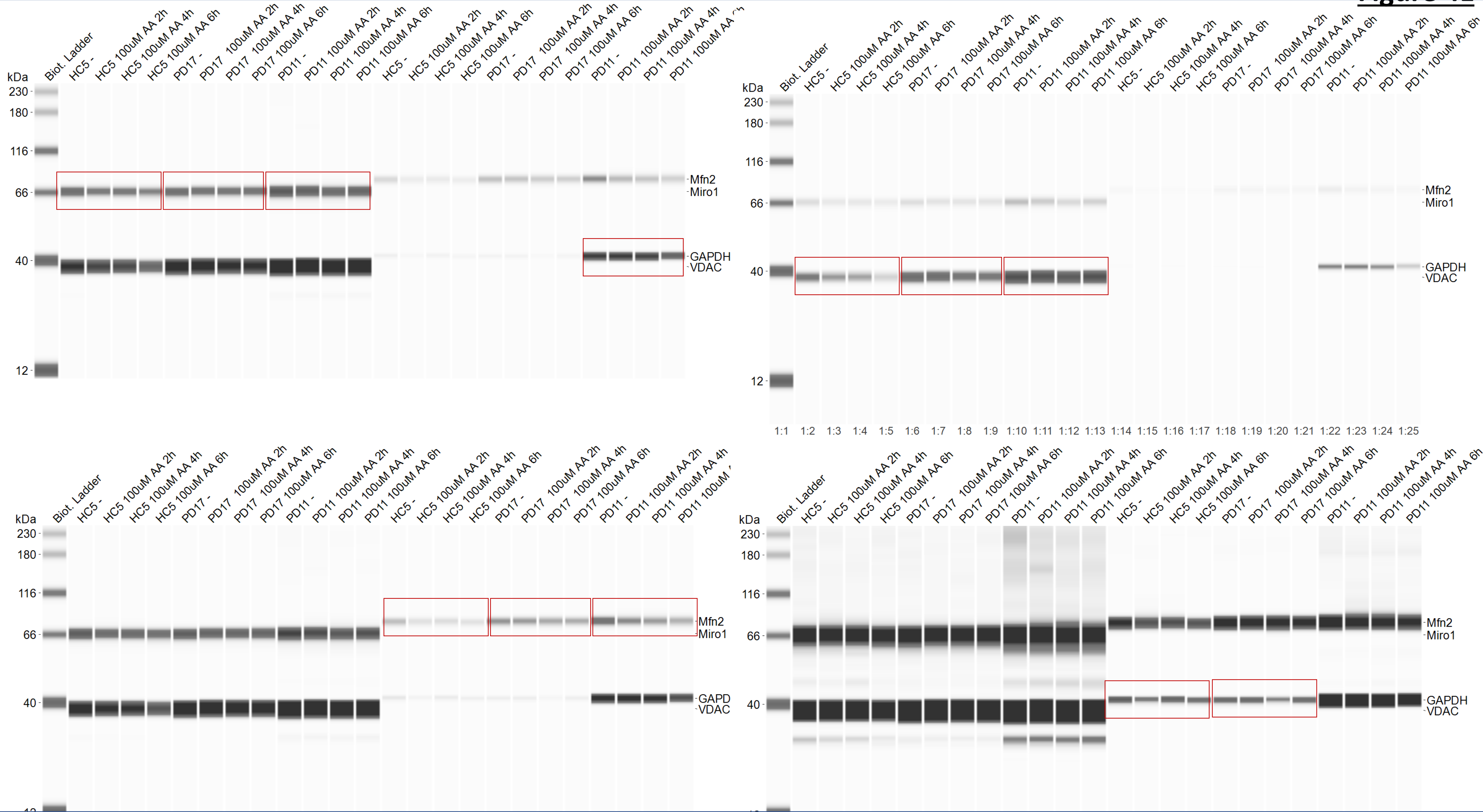
kDa
230
180
116
66
40
12
Biot. Ladder
HC5 -
HC5 100uM AA 2h
HC5 100uM AA 4h
HC5 100uM AA 6h
PD17 -
PD17 100uM AA 2h
PD17 100uM AA 4h
PD17 100uM AA 6h
PD11 -
PD11 100uM AA 2h
PD11 100uM AA 4h
PD11 100uM AA 6h
Mfn2
Miro1
GAPDH
VDAC
1:1 1:2 1:3 1:4 1:5 1:6 1:7 1:8 1:9 1:10 1:11 1:12 1:13 1:14 1:15 1:16 1:17 1:18 1:19 1:20 1:21 1:22 1:23 1:24 1:25

Figure5 A

HC2, PD4, PD6

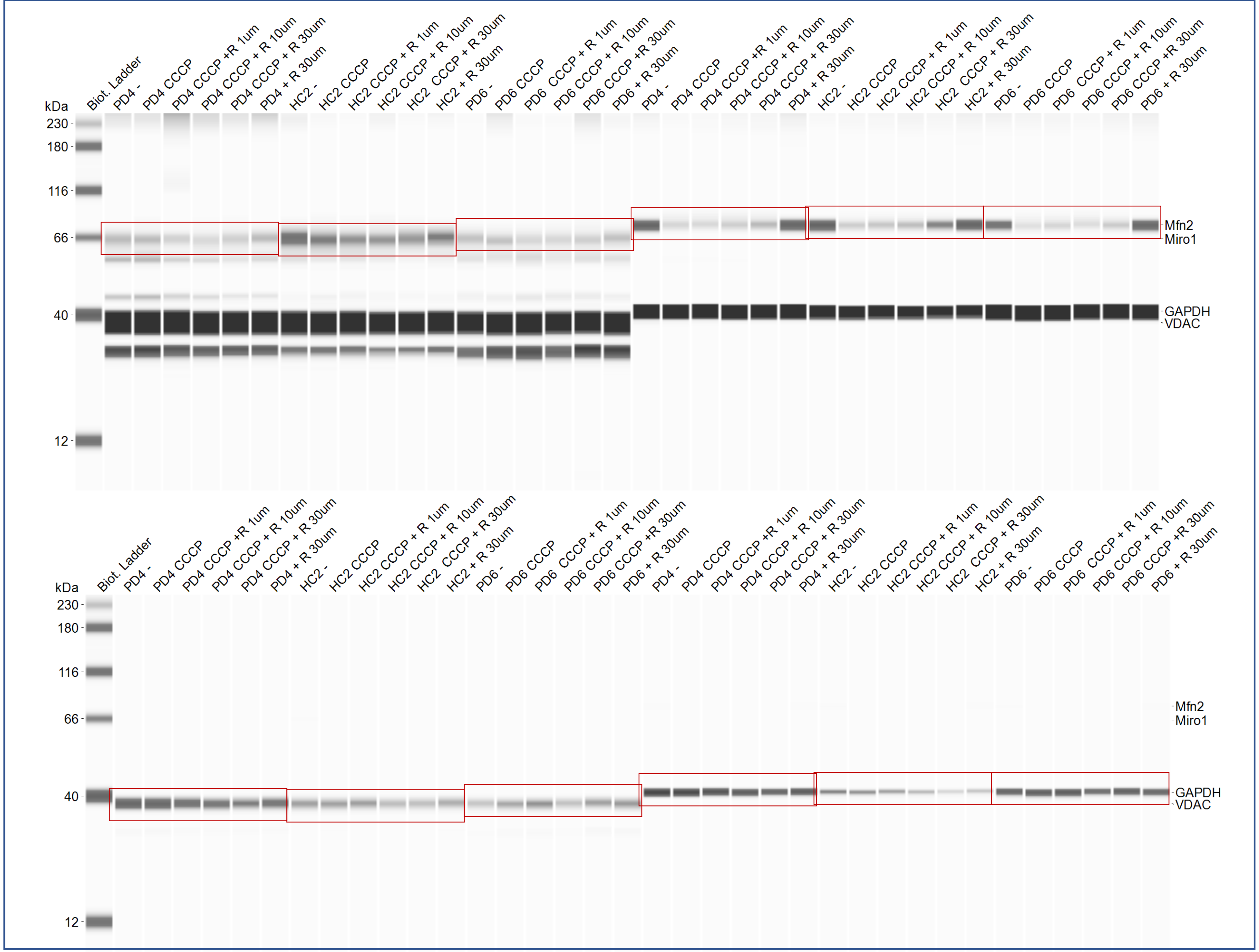

Figure5 A

HC1, PD1, PD5

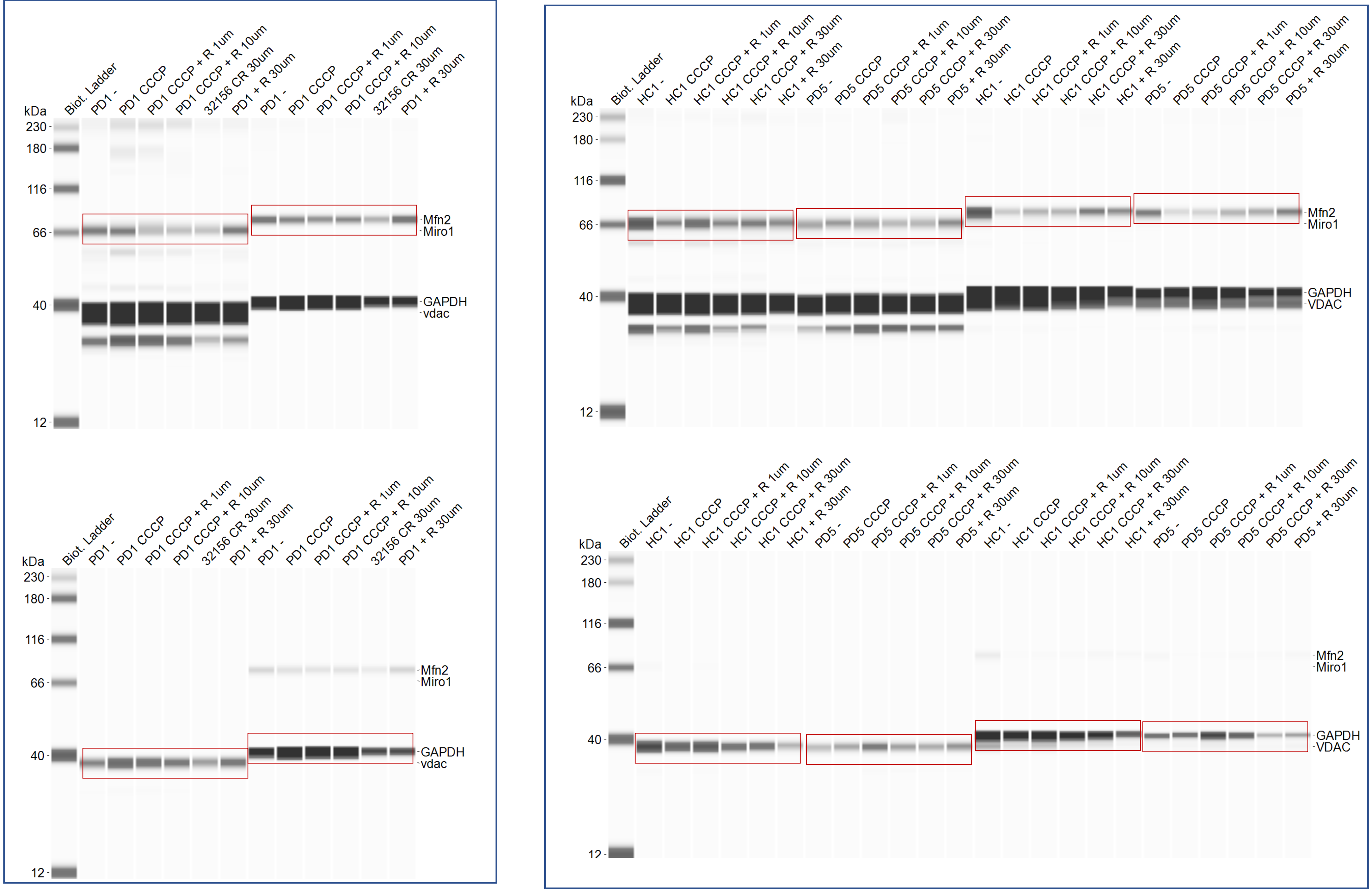

Supplement: Supplementary file 3 — Supplementary figures 1 and 2 [file 41531_2025_1115_MOESM3_ESM.pdf]
